# Economic Value of Data and Analytics for Health Care Providers: Hermeneutic Systematic Literature Review

**DOI:** 10.2196/23315

**Published:** 2020-11-18

**Authors:** Philip von Wedel, Christian Hagist

**Affiliations:** 1 Chair of Economic and Social Policy WHU - Otto Beisheim School of Management Vallendar Germany

**Keywords:** digital health, health information technology, healthcare provider economics, electronic health records, data analytics, artificial intelligence

## Abstract

**Background:**

The benefits of data and analytics for health care systems and single providers is an increasingly investigated field in digital health literature. Electronic health records (EHR), for example, can improve quality of care. Emerging analytics tools based on artificial intelligence show the potential to assist physicians in day-to-day workflows. Yet, single health care providers also need information regarding the economic impact when deciding on potential adoption of these tools.

**Objective:**

This paper examines the question of whether data and analytics provide economic advantages or disadvantages for health care providers. The goal is to provide a comprehensive overview including a variety of technologies beyond computer-based patient records. Ultimately, findings are also intended to determine whether economic barriers for adoption by providers could exist.

**Methods:**

A systematic literature search of the PubMed and Google Scholar online databases was conducted, following the hermeneutic methodology that encourages iterative search and interpretation cycles. After applying inclusion and exclusion criteria to 165 initially identified studies, 50 were included for qualitative synthesis and topic-based clustering.

**Results:**

The review identified 5 major technology categories, namely EHRs (n=30), computerized clinical decision support (n=8), advanced analytics (n=5), business analytics (n=5), and telemedicine (n=2). Overall, 62% (31/50) of the reviewed studies indicated a positive economic impact for providers either via direct cost or revenue effects or via indirect efficiency or productivity improvements. When differentiating between categories, however, an ambiguous picture emerged for EHR, whereas analytics technologies like computerized clinical decision support and advanced analytics predominantly showed economic benefits.

**Conclusions:**

The research question of whether data and analytics create economic benefits for health care providers cannot be answered uniformly. The results indicate ambiguous effects for EHRs, here representing data, and mainly positive effects for the significantly less studied analytics field. The mixed results regarding EHRs can create an economic barrier for adoption by providers. This barrier can translate into a bottleneck to positive economic effects of analytics technologies relying on EHR data. Ultimately, more research on economic effects of technologies other than EHRs is needed to generate a more reliable evidence base.

## Introduction

Data and analytics applications increasingly find their way into our health care systems. Some manifestations of data, like the electronic health record (EHR), have already been more established in many member countries of the Organisation of Economic Co-operation and Development. Analytics technologies such as computerized clinical decision support (CCDS) or advanced analytics (AA) based on big data and artificial intelligence (AI) still seem to be newcomers in this field. Hopes are high that data and analytics significantly improve quality, efficiency, and patient experience of health care delivery [1]. Taking the perspective of health care systems, latest research, indeed, shows that adoption of EHRs leads to fewer medication errors, less adverse drug effects, and higher guideline adherence [2,3]. The use of clinical decision support (CDS) tools is associated with lower morbidity, potentially improving mortality [4,5]. Based on EHR data, AA is already able to predict the onset of several diseases like diabetes, schizophrenia, or cancer as well as provide care-related forecasts of in-hospital mortality, unplanned readmissions, length of stay, or infection risks [6-9]. One of the more recent topics is the possibility to diagnose the novel coronavirus disease (COVID-19) by applying AI to chest computed tomography scans [10,11]. It becomes clear how the introduction of these technologies can clearly create positive spillover effects for the entire health care system. When taking the microperspective of single providers, however, current adoption of data and analytics seems to paint a different picture in many countries. In the United States or Denmark, almost all hospitals work with a sophisticated EHR, while many European countries show much lower adoption rates. For example, reports indicate that 38.3% to 47.4% of German or 27.8% to 46.4% of Austrian hospitals lacked a system entirely in 2017 [12,13]. Even though analytics applications relying on AI or big data show strong potential, adoption in everyday provider operations is still comparatively low [14]. The reasons for this are manifold and include social, ethical, legal, or technological barriers [15]. The most powerful barrier, however, is still of an economic nature. Health care providers see the initial and ongoing maintenance costs as key barriers for adoption and oftentimes question overall cost-effectiveness of these solutions [15-17]. In a world that has not yet significantly pivoted towards quality-based reimbursement, quality improvements via data and analytics are, ironically, not necessarily directly linked to economic benefits for single health care providers. The much higher adoption of EHRs in the United States can, to a large part, be explained by strong financial subsidies by policy makers [18,19]. Taking the single provider’s perspective, the question pertains whether hospitals, clinics, and practices can gain economic benefits from the usage of data and analytics. Most existing reviews in this field heavily focus on EHRs, but do not take into consideration other analytics tools [20-23]. Other more recent reviews focus on the economic impact of single areas of data and analytics like AI, but not specifically on providers [24]. Our work attempts to fill this gap by providing a comprehensive review of the literature covering the economic impact of several applications of data and analytics exclusively on providers. In the end, the promising potential of a number of established and rapidly evolving technologies to improve quality of care in our health care systems can only be optimally leveraged via widespread adoption by single providers.

## Methods

### Hermeneutic Systematic Review

The common systematic review ideally represents a highly structured approach for searching, screening, including, and summarizing studies to answer a rather narrowly defined question [25,26]. It might not, however, show perfect fit with all research questions. As Greenhalgh et al [27] summarized, it often “can be viewed as a set of methodologies characterized by tight focus, exhaustive search, high rejection-to-inclusion ratio and an emphasis on technical rather than interpretive synthesis methods.” The hermeneutic review methodology introduced by Boell and Cecez-Kecmanovic [28] showed a particularly good fit with the broader nature of this study’s research question. This process of a literature review follows 2 interlinked cycles: (1) search and acquisition and (2) analysis and interpretation (see Multimedia Appendix 1). The hermeneutic process allows and encourages a constant process of refining and extending the search realm of cycle (1) by deeply engaging with the content of the identified literature via cycle (2). This enables the researcher to leverage “the importance of reading and dialogical interaction between the literature and the researcher, […] seeking originality rather than replicability” [28]. Nevertheless, to assure the systematic execution of this review, guidelines for scoping studies including a 6-step process by Arksey and O’Malley [29] were followed. The hermeneutic approach was hereby complemented by the established tools for study identification and charting, assuring a systematic execution of the review. These tools resulted in a clear, reproducible, and structured overview of how studies were identified and for which reasons studies were excluded. In the end, by combining the traits of systematic and hermeneutic reviews, this study attempted to generate a structured, reproducible, comprehensive, and content-focused review of the literature.

### Search Strategy

Literature included in this review was identified via iterative structured keyword searches in the online databases PubMed and Google Scholar, as well as a complementary backward and manual search. The following keyword search on article titles was applied to both databases: (*x*) AND (cost(s) OR revenue OR benefit OR return OR ROI OR value OR efficiency OR productivity) AND (hospital(s) OR practice(s) OR provider(s)). In this search, *x* represented a placeholder for terms that were iteratively added following the hermeneutic approach, and the following segments assured inclusion of studies only covering economic effects for only health care providers and which remained unchanged for all searches. In an initial search, *x* was comprised of “Electronic Health Record,” “Electronic Medical Record,” “Electronic Patient Record,” “Analytics,” and “Clinical Decision Support” (including all alternative and plural types of wording and abbreviations). Following the hermeneutic approach, both authors independently screened the resulting studies and jointly decided on additional search terms, expanding *x* to also include the terms “Algorithm,” “Artificial Intelligence,” “Big Data,” “Machine Learning,” “Deep Learning,” “Natural Language Processing,” and “Telemedicine.” Interestingly, searches in the field of mobile health (mHealth) including health applications did not generate any suitable results. The search was limited to journal articles published in English between January 2009 and December 2019. The exact search queries for both databases can be found in Multimedia Appendix 2.

## Results

### Study Selection

**Figure 1:**
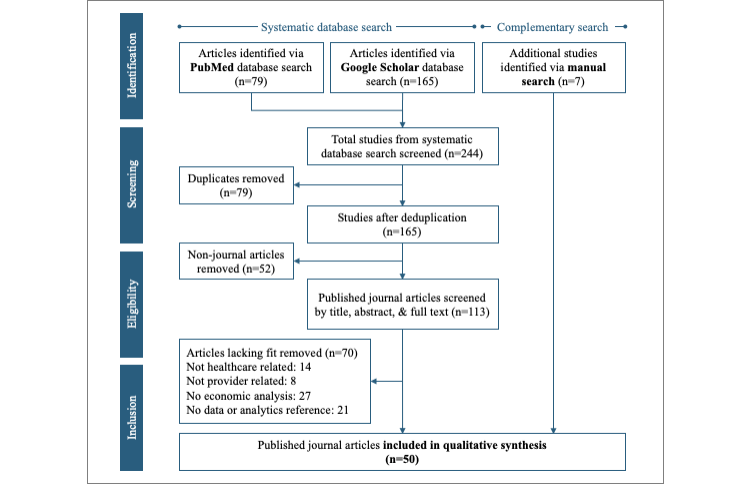
Literature search and study selection process.

The PubMed and Google Scholar searches generated 79 and 165 results, respectively (see Figure 1). Following deduplication, a total of 165 studies remained for more detailed review. Only published journal articles were considered for the review. Hence, the deduplicated search results were again cleaned, resulting in 113 articles. Titles, abstracts, and, if needed, content of these articles were analyzed by both authors independently in order to determine fit to the research question, narrowing results to 43 results. Frequent reasons for exclusion were articles dealing with effects of data and analytics on stakeholders other than providers or on overarching national health care spending. Other examples were articles covering analogue tools and processes like paper-based decision support or diagnostic testing decision algorithms. An overview of all 113 screened studies and respective reasons for exclusion can be found in Multimedia Appendix 3. A complementary backward and manual search by both authors independently resulted in an additional 7 articles for inclusion. The final 50 articles were thoroughly reviewed, and key properties were summarized by the first author in a structured manner to facilitate pattern identification and final synthesis generation (see Multimedia Appendix 4).

### Study Categorization

**Figure 2:**
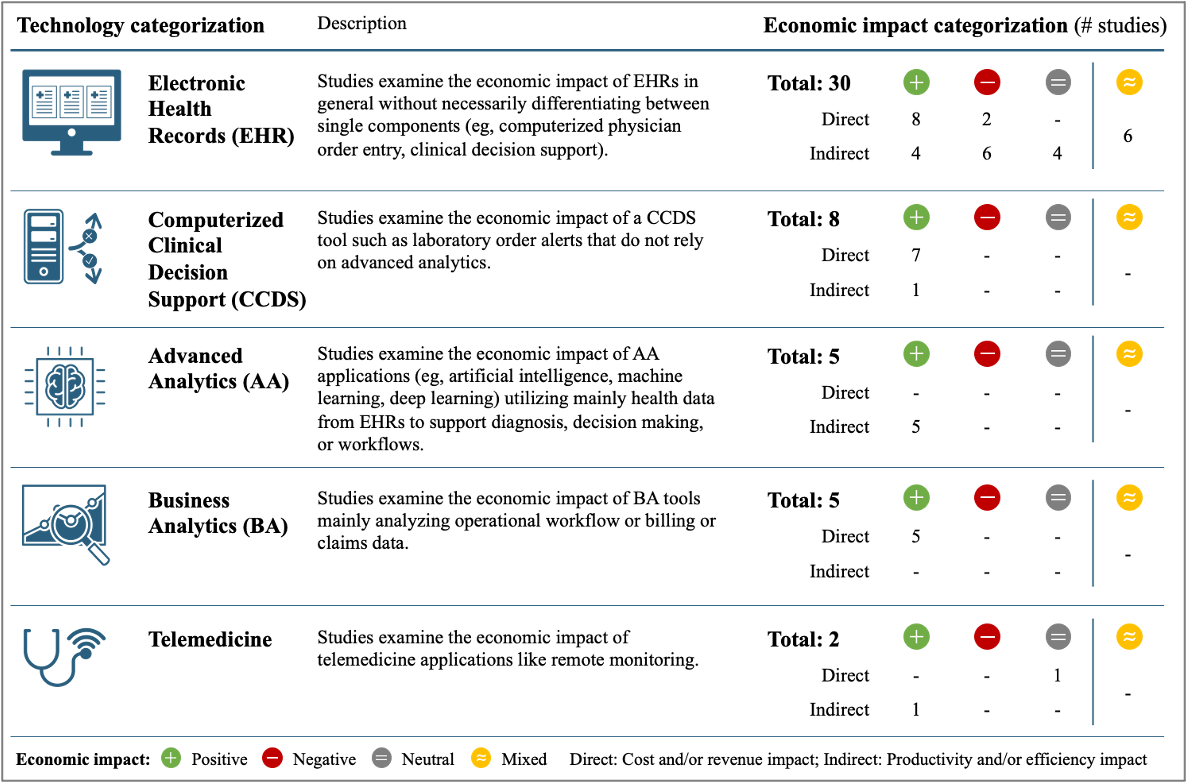
Overview of reviewed studies categorized by technology and economic impact.

Following in-depth review of the 50 final studies, 2 angles for categorization emerged. First, the studies were sorted according to the technology under research, resulting in 5 key categories, namely EHRs, CCDS, AA, business analytics (BA), and telemedicine. Second, studies were categorized based on the type of identified economic impact. This impact categorization consists of 2 combined components, namely mode (direct vs indirect) and direction (positive vs negative vs neutral vs mixed). Considering the impact mode, studies were categorized to have an *indirect* impact when no *direct* impact on costs or revenue but on efficiency or productivity was shown (see Figure 2 for a summary and details).

### Electronic Health Records

At 60% (30/50) of identified articles, EHRs represented the most comprehensive body of literature by far. In terms of economic impact, overall, a rather ambiguous pattern could be observed, with 12 studies revealing a positive, 8 studies a negative, 4 studies a neutral, and 6 studies a mixed economic effect on providers. The majority of studies was US-based (20/50, 40%), with Asia (3/60, 5%), the rest of the world (2/60, 3%), and Europe (0/60) showing less research activity. The remaining 5 articles represented international literature reviews.

The 5 literature reviews included in the sample predominantly indicated mixed economic impacts of EHRs. All reviews included studies proving positive effects mostly via increased efficiency; however, for almost every review, another identified article indicated the opposite [21-23,30]. Only Highfill [20] revealed overall positive economic effects, determining a 1.1%-13.8% cost decrease (95% CI) after EHR introduction in their meta-analysis.

Cost-benefit analyses (CBA) were presented in 5 articles and also painted a slightly ambiguous picture, with significantly varying timelines for EHR installations to break even. The majority of studies indicated an average breakeven timeline between 3 and 8 years for EHR implementations in hospitals [31-33]. Jang et al [34] indicated a much shorter 6.2-17.4–month (95% CI) breakeven timeframe for primary care clinics. Only 1 study revealed a clear negative impact, showing a negative 5-year return on investment [35]. In general, the CBAs provided some interesting practice-oriented insights for EHR implementations. Results from Choi et al [31] and Adler-Milstein et al [35] both emphasized the importance of fully eliminating legacy costs like paper-based records and related dictation services. Parallel digital and analog structures resulted in fewer efficiency gains and, hence, longer breakeven timelines. Besides decreasing costs, a successful EHR introduction also focused on additional revenue generation via improved charge capture and reduction of billing errors [31,32,35]. Lastly, Jang et al [34] showed that more recent EHR systems and those using flow diagrams also came with shorter breakeven timelines, implying potential important technological advances by vendors over the years.

Besides the full CBAs, 6 studies examined the effects of EHR introductions on a variety of single-cost or revenue items. Encinosa and Bae [36] showed how the introduction of advanced EHRs reduced adverse drug effects from 3.6% to 1.4% of all cases, saving an average of US $4790 per avoided case. Joseph [37] revealed how personnel formerly needed for paper-based record keeping could be reduced, thereby saving more than US $6 million over 5 years. Zlabek et al [38] showed how transcription costs were significantly reduced, resulting in US $667,896 in costs saved 1 year after EHR introduction. A different source of cost savings was the avoidance of redundant laboratory tests and imaging exams. A computerized physician order entry (CPOE) system within an EHR resulted in an 18% decrease in laboratory test orders, as well as 6.3% fewer radiology exams [38,39]. However, Schnaus et al [40] revealed the importance of appropriate execution of a CPOE implementation. The authors examined a temporary change regarding the preselected laboratory test type when physicians searched for a complete blood count (CBC) within the CPOE tool. For 23 days, the system preselected a slightly more costly version of a CBC. Presumably due to time constraints, a number of physicians did not double-check this preselected test type, which resulted in an average daily cost increase for CBC testing of US $293.10. Besides the direct economic impact via costs, some studies demonstrated positive effects via revenue. Terry [41] highlighted the potential from value-based reimbursement based on EHR data. The author saw the lack of an EHR system as “an ‘opportunity cost’ that can be quantified and weighed against the cost of installing a system” [41].

Finally, a significant share of papers (14/30) examined indirect economic impacts of EHRs via changes in efficiency or productivity. Here, a rather negative image emerged, with only 4 studies revealing positive effects, and the remaining showing either negative (6/30) or neutral (4/30) effects. Due to significant heterogeneity, it was difficult to draw generalizable insights from this sample of literature. This was a takeaway the identified systematic reviews also revealed. 5 studies examined productivity changes after EHR introduction, where productivity was mostly defined as average patient volumes. Of these studies, 3 revealed no statistically significant changes, hence neutral economic impacts [42-44]. Kaneko et al [45] showed a negative impact on multifactor productivity following EHR introduction in Japanese municipal hospitals. Only 1 study revealed positive long-term effects on productivity [46]. In 9 studies, efficiency implications were examined, where efficiency was defined rather heterogeneously as treatment times, waiting times, length of stay, or personnel volumes. While 1 study showed no effects [47], 5 studies revealed a negative economic impact [48-52]. Especially, the implementation of a fully-fledged EHR in a relatively short period of time, a so-called “big bang” introduction, seemed to be detrimental to hospital efficiency [51]. Only 3 studies showed somewhat limited positive effects on provider efficiency following EHR implementation [53-55].

### Computerized Clinical Decision Support

Studies examining the economic impact of CCDS on providers represented the second-largest share of identified articles, at 16% (8/50). A strong picture regarding the impact emerged, with all 8 studies revealing a positive economic impact on providers, predominantly of a direct nature. Again, the majority of articles was US-based (5/8, 63%), and others were located in the rest of the world (2/8, 30%). The remaining article represented an international literature review.

Bright et al [5] presented the only included systematic review of CDS tools also assessing their impact on costs. Of a total 148 identified papers, 22 studies analyzed costs, of which 13 implied cost reductions. The authors saw this as “modest evidence from academic and community inpatient and ambulatory settings” [5]. Not all included studies, however, examined fully computerized CDS tools.

In 3 articles, it was shown how CCDS systems could reduce the number of imaging studies, laboratory tests, or the amount of medicine utilized. Fleddermann et al [56] assessed the introduction of an automated alert to avoid unnecessary ordering of echocardiography studies. Over the study period, 20% of the respective studies were cancelled, thereby saving the associated costs. Okumura et al [57] examined the cost savings associated with implementing a tool to optimize antibiotic use in surgical prophylaxis. By reminding physicians of common standards of care, the system decreased the usage significantly by 1.26 defined daily doses per 100 bed days to –0.2 defined daily doses per 100 bed days (95% CI), thereby saving an estimated US $50,000 per 100 bed days. Lastly, Levick et al [58] assessed an alert for B-type natriuretic peptide testing. Again, the alert resulted in a test reduction of 21%, saving an estimated US $92,000 per year.

Besides effects via reduced volumes in tests or studies, 3 other articles revealed cost savings via supporting decisions regarding care processes and workflows. Quadros et al [59] examined CDS that supported fast tracking the discharge of certain patients after brain tumor surgery. The tool resulted in a significant length of stay reduction of 2 days on average, saving US $630 per hospitalization. Collins et al [60] showed how decisions on the timing of nasal feeding tube insertions for poststroke patients with dysphagia supported by CDS reduced the number of nasal tube replacements and repeat x-rays and the associated costs. It is important to mention here, however, that these 2 papers did not reveal whether the CDS tools were fully computerized. Lastly, Wagholikar et al [61] presented the impact of a CCDS tool in an outpatient setting. Here, the tool supported physicians with chart review via a computerized checklist to decide on preventive services and management of chronic diseases. The tool showed an indirect positive economic impact by reducing review times by 65% per patient.

The eighth article in the CCDS category by Elkin et al [62] is the only one examining direct cost savings based on supporting diagnosis. The authors applied a differential diagnosis support tool to cases in diagnostically challenging Diagnostic Related Groups and found that, for these patients, the provider costs per case were reduced by 3.7%, to 19.5% (95% CI).

### Advanced Analytics

The recently increasingly prominent field of AA including AI, machine learning, and deep learning represented only 10% of the identified literature (5/50). A very homogenous picture was painted, with all 5 studies indicating indirect positive economic effects on providers; 3 articles were US-based, and others originated from Europe (1/5) and Asia (1/5).

The identified articles showed 2 main use cases of AA. First, 2 articles showed how AA could support decision making in the field of imaging. Lee [63] applied a convolutional neural network to determine musculoskeletal magnetic resonance imaging scanning protocols. The authors hypothesized that this assistance in protocol generation could potentially save personnel time and hence improve provider efficiency. The second article presented the deployment of the IBM Watson natural language processing model to automatically decide on the usage of intravenous contrast for magnetic resonance imaging protocols [64]. Again, the authors hypothesized that this support in decision making has the potential to drive provider efficiency.

The second use case represented the prediction of patients’ disease progression and the associated care processes. Wang et al [65] showed how a convolutional neural network–based tool using hospital EHR data could predict readmissions. Readmission predictions can be valuable information since a majority of readmissions is associated with penalties for providers. Nevertheless, the authors only hypothesized this potential benefit. Almeida [66] presented a case study of a hospital center in Portugal that applied a big data analytics platform. Based on EHR and vital sign data, the system was able to correctly predict 30% of intensive care unit admissions and 50% of non-intensive care unit inpatient deaths. Again, the author hypothesized potential efficiency improvements. Lastly, Peck et al [67] represented the only article in the AA category, which proved actual efficiency improvements instead of only hypothesizing them. The authors presented the impact of a tool predicting the patient flow from the emergency department to the inpatient units via discrete event simulation. By sharing information on crowding levels and total expected beds needed with physicians and nurses, the boarding time from the emergency department to inpatient units was reduced by between 11.69% and 18.38%, depending on hospital type.

### Business Analytics

Studies examining the economic impact of BA on providers represented 10% of identified articles (5/50). All 5 studies revealed a positive economic effect on providers, mostly of a direct nature. The majority of articles represented US-based (4/5) or Europe-based (1/5) case studies.

BA tools analyzing equipment utilization were examined in 2 articles. Stekel et al [68] examined the example of an ultrasound practice that used probe utilization data to support purchasing decisions. The analysis of procedure data resulted in the decision to not replace a broken probe, thereby saving US $10,000. Swedberg [69] showed how attaching radiofrequency identification tags to all equipment in a 1100-bed hospital increased equipment utilization rates from 5% to 40%. The system was able to reduce the need to rent or purchase additional equipment, saving an estimated US $200,000 per year.

Examples of how BA applications improved billing by reducing revenue leakage or avoiding penalties were presented in 3 articles [70-72]. For example, Dulac et al [70] showed in a case study how a US-based hospital used data analytics to uncover root causes for an increase in preventable complications and readmission rates that implied payment reductions totaling 3.5% of total revenue. The tool helped the hospital to ultimately reduce penalties to 0%.

### Telemedicine

Studies examining the economic impact of telemedicine on providers represented the smallest share of identified articles (2/50). Both articles originated from Europe, with 1 study revealing an indirect positive effect and 1 a neutral effect.

Stoves et al [73] examined the advantages of an electronic medical round connecting general practitioners with nephrologists in the field of chronic kidney care. The program was perceived to improve efficiency for both physicians and nephrologists, as indicated in interviews and questionnaires; however, no quantitative efficiency improvements were reported. Heidbuchel et al [74], on the other hand, specifically analyzed differences in costs and financial impact between remote and in-office follow-ups for implantable cardiac defibrillators. On average, the total cost and net financial impact for providers were neutral, not showing differences between remote and in-office follow-ups. Importantly, however, regional heterogeneity could be observed where providers in countries with remote follow-up reimbursement in places like Germany maintained or improved economics.

## Discussion

### Principal Findings

At first indication, the presented results appear to generate an overall positive answer to the overarching research question of the economic impact data and analytics have on health care providers. Of the 50 reviewed articles, 31 indicated a positive impact either via direct cost or revenue effects or via efficiency or productivity improvements. Studies showed how EHRs can, for example, directly save storage and personnel costs associated with paper records or increase physician productivity by making information available when and where it is needed. Other studies proved that CCDS can save material and labor costs by avoiding redundant laboratory tests and imaging studies. A more nuanced look at the results, however, shows that it is very important to differentiate between the 5 identified technology categories. In line with other literature reviews, a mixed overall picture, at best, was revealed for the economic impact of EHRs, or “data,” on providers. From a provider’s perspective, 12 studies revealing a positive result and 18 revealing negative, neutral, or mixed results do not necessarily promote a quick decision on EHR investments, at least from an economic point of view. On the other hand, “analytics” applications like CCDS, AA, and BA seem to predominantly generate positive economic effects. Nevertheless, the small number of identified papers covering these technologies, yet again, points at the risk of discouraging rapid adoption by providers from an economic point of view. Ultimately, this review reveals a rather uncomfortable decision-making situation for providers with the economic impact of “data,” represented by EHRs, being exhaustively researched but revealing ambiguous results and “analytics” indicating positive results but being only sparsely investigated.

Considering the positive effects of EHRs on health care outcomes, the identified ambiguous results regarding the economic impact for providers also implies potentially missing out on the associated welfare gains across populations. Some nations’ policy makers already acknowledged this and incentivize EHR adoption. The United States Health Information Technology for Economic and Clinical Health Act from 2009 injected several billions of dollars into the system for subsidized EHR installations [19]. This approach seemed to have worked as intended by pushing EHR adoption closer to 100% for US hospitals [18]. Of course, national health systems strongly vary, but this outcome should at least foster a discussion of whether EHR subsidization might also be a solution in other countries with comparably low current EHR adoption rates. Germany, for example, announced a “hospital future law” (Krankenhauszukunftsgesetz) as part of a COVID-19 stimulus package in 2020, which envisions an up to €4.3 billion fund for investments in digital infrastructures and emergency capacities [75]. Here, it is important to point out the positive spillover effects of data and analytics for the entire health care system again. Even though this review predominantly takes a microperspective of the single provider, in the end, adoption on the microlevel is a key prerequisite for changes or improvements at the system level. Data and analytics might provide proven positive effects on quality of care, but until the world does significantly pivot towards quality-based care and reimbursement, alternative ways to foster technology adoption should be considered.

Another effect involving EHRs is also important to consider. Several of the included studies showed how EHR installations can act as a door-opener to other technologies that actually seem to predominantly have positive economic effects for the provider. Especially, 2 other technology categories identified in this review, namely CCDS and AA, strongly rely on data contained in EHRs. More precisely, 75% (6/8) of the CCDS tools and 60% (3/5) of the AA tools in the identified studies needed some sort of EHR input. Even if somewhat limited in quantity, the current identified research in these 2 fields revealed only positive economic effects for providers. Hence, EHR adoption can become a bottleneck to the positive economic effects of technologies further down the line like CCDS and AA. Following EHR adoption, providers are likely able to derive economic benefits from adjoined technologies identified in this review. More research dedicated to these economic effects of supplementing EHRs with adjoined technologies like CCDS and AA is needed to derive a more targeted evidence base.

Leaving policy implications aside, our work generates insights for providers as well. For providers considering an EHR installation, this review showed important factors for an economically feasible introduction. Eliminating all legacy costs like paper-based records and related dictation services, repurposing paper record space into clinical space, or installing new technology in a stepwise fashion (avoiding a big bang) are all important takeaways from this review. For hospitals or practices already using an EHR, adjacent technologies, like CCDS or AA, can provide economic benefits, potentially even resulting in a shorter breakeven time for the EHR installation. Additionally, the emerging opportunities to participate in value-based care plans utilizing EHR data or the utilization of business intelligence should not be fully neglected. Nevertheless, a number of other potential sources of economic value from data seems not to be currently covered by research. For example, no study was identified that covered the potential for direct monetization of anonymized patient data or the ability to drive patient volumes by marketing the application of advanced digital tools.

Ultimately, it is important to note that research on the economic impact of data and analytics on providers remains rather limited in geographies other than the United States and in technologies other than EHR. In general, this review did not identify geography as a predictor for the type of economic impact. However, with almost 65% (32/50) of included articles being US-based, more research in other geographies is needed to draw a definite conclusion whether geographies and related health care systems are significant drivers. From a technology perspective, the few studies covering technologies other than EHR revealed proof for economic advantages; however, no comprehensive cost-benefit analyses and few systematic reviews were identified for these technologies. In the field of AA, 80% (4/5) of identified studies only hypothesized economic benefits. In the near future, however, vendors of AA tools need to also provide high-quality proof for the economic advantages of their solutions.

### Limitations

This work is exposed to limitations that are mostly inherent to literature reviews in general. Only PubMed and Google Scholar online databases were searched; hence, relevant research captured exclusively by other databases could have been excluded. The sample of identified articles potentially lacks certain avenues of research not captured by the structured keyword search, thereby missing other technologies. The applied hermeneutic systematic search approach, however, worked against these limitations by explicitly allowing for iterative searches. Additionally, the systematic search was complemented by manual search techniques. On a different note, most identified studies are based in the United States; hence, conclusions might not be fully applicable to other geographies. Ultimately, it is important to note that the research subject “data” is almost exclusively represented by studies focusing on EHRs, thereby not touching on other potentially relevant sources and applications of data. Nevertheless, EHRs can be considered as a key data container in the context of health care. The research subject “analytics,” on the other hand, faces a very limited body of evidence, which strongly impacts the generalizability of this study’s findings. More research covering these other technologies is needed to generate a more holistic and reliable evidence base. Lastly, the intended broad spectrum of reviewed studies prevents a clear and uniform definition and quantification of “economic value.” Studies and respective results can, hence, not be compared on the same scale, also since the methodological quality of the original studies was not analyzed.

### Conclusion

This review synthesized literature examining the economic value of data and analytics for health care providers. Five key technologies were identified, namely EHRs, CCDS, AA, BA, and telemedicine. Overall, 31 of the 50 reviewed articles indicated a positive economic impact, either via direct cost or revenue effects or via efficiency or productivity improvements. A more nuanced view showed that this is especially the case for less studied technologies like CCDS, AA (including AI and big data analysis), and BA. For the most extensively studied technology of EHRs, a more ambiguous view with varying economic impacts emerged. Since technologies like CCDS and AA strongly rely on EHR data, these ambiguous research findings have the potential to turn EHR adoption into a bottleneck for the adjoined technologies with mostly positive economic effects. This review also encourages discussions around how subsidization of EHRs, like that implemented in the United States and planned for Germany, could potentially unlock the proven economic potential of second-order adjoined technologies. It can be concluded that more research covering the economic effects of technologies other than EHRs would significantly improve the current evidence base and potentially drive adoption by health care providers.

## Appendix

Multimedia Appendix 1 The interlinked cycles of the hermeneutic process.

Multimedia Appendix 2 Detailed search queries used for the PubMed and Google Scholar database searches.

Multimedia Appendix 3 Overview of all published articles screened by title, abstract, and text (including reasons for exclusion).

Multimedia Appendix 4 Detailed syntheses of the final 50 studies included in this review.

## Abbreviations

AA: advanced analytics
AI: artificial intelligence
BA: business analytics
CBA: cost-benefit analysis
CBC: complete blood count
CCDS: computerized clinical decision support
CDS: clinical decision support
CPOE: computerized physician/provider order entry
EHR: electronic health record
