# Supplementary figures and images for: Economic Value of Data and Analytics for Health Care Providers: Hermeneutic Systematic Literature Review
Source: J Med Internet Res. 2020 Nov 18;22(11):e23315. doi: 10.2196/23315 (PMC7710451; doi:10.2196/23315)

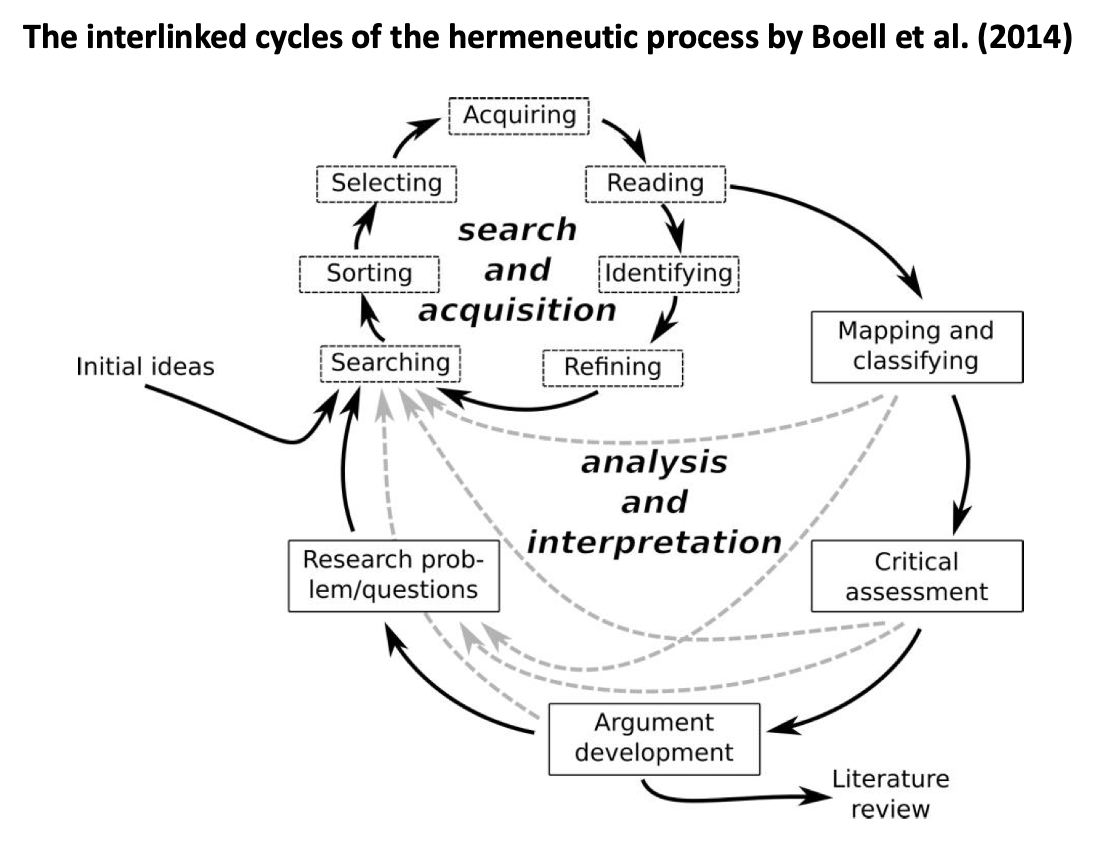

Supplement: Multimedia Appendix 1 [file jmir_v22i11e23315_app1.png]
