# Supplementary material for: Economic Value of Data and Analytics for Health Care Providers: Hermeneutic Systematic Literature Review
Source: J Med Internet Res. 2020 Nov 18;22(11):e23315. doi: 10.2196/23315 (PMC7710451; doi:10.2196/23315)
Supplement: Multimedia Appendix 2 [file jmir_v22i11e23315_app2.pdf]

Multimedia Appendix 2: Systematic database search

Google Scholar (01.01.2009 – 31.12.2019)

EHR (n=39)
allintitle: (EHR OR "electronic health record" OR "electronic health records") (cost OR costs OR revenue OR benefit OR return OR ROI OR value OR efficiency OR productivity) (hospitals OR hospital OR practices OR practice OR provider OR providers)

EMR (n=35)
allintitle: (EMR OR "electronic medical record" OR "electronic medical records") (cost OR costs OR revenue OR benefit OR return OR ROI OR value OR efficiency OR productivity) (hospitals OR hospital OR practices OR practice OR provider OR providers)

EPR (n=1)
allintitle: (EPR OR "electronic patient record" OR "electronic patient records") (cost OR costs OR revenue OR benefit OR return OR ROI OR value OR efficiency OR productivity) (hospitals OR hospital OR practices OR practice OR provider OR providers)

Analytics (n=22)
allintitle: (analytics) (cost OR costs OR revenue OR benefit OR return OR ROI OR value OR efficiency OR productivity) (hospitals OR hospital OR practices OR practice OR provider OR providers)

AI & Big Data (n=19)
allintitle: (AI OR "artificial intelligence" OR "big data") (cost OR costs OR revenue OR benefit OR return OR ROI OR value OR efficiency OR productivity) (hospitals OR hospital OR practices OR practice OR provider OR providers)

Algorithms, ML & DL (n=31)
allintitle: (algorithm OR algorithms OR "machine learning" OR “deep learning”) (cost OR costs OR revenue OR benefit OR return OR ROI OR value OR efficiency OR productivity) (hospitals OR hospital OR practices OR practice OR provider OR providers)

Decision Support (n=18)
allintitle: ("decision support") (cost OR costs OR revenue OR benefit OR return OR ROI OR value OR efficiency OR productivity) (hospitals OR hospital OR practices OR practice OR provider OR providers)

NLP (n=0)
allintitle: ("natural language processing" OR NLP) (cost OR costs OR revenue OR benefit OR return OR ROI OR value OR efficiency OR productivity) (hospitals OR hospital OR practices OR practice OR provider OR providers)

Telemedicine (n=0)
allintitle: (telemedicine) (cost OR costs OR revenue OR benefit OR return OR ROI OR value OR efficiency OR productivity) (hospitals OR hospital OR practices OR practice OR provider OR providers)

Σ n=165

PubMed (01.01.2009 – 31.12.2019)

EHR (n=28)
(((EHR[Title] OR "electronic health record"[Title] OR "electronic health records"[Title])) AND (cost[Title] OR costs[Title] OR revenue[Title] OR benefit[Title] OR return[Title] OR ROI[Title] OR value[Title] OR efficiency[Title] OR productivity[Title])) AND (hospitals[Title] OR hospital[Title] OR practices[Title] OR practice[Title] OR provider[Title] OR providers[Title])

EMR (n=16)
(((EMR[Title] OR "electronic medical record"[Title] OR "electronic medical records"[Title])) AND (cost[Title] OR costs[Title] OR revenue[Title] OR benefit[Title] OR return[Title] OR ROI[Title] OR value[Title] OR efficiency[Title] OR productivity[Title])) AND (hospitals[Title] OR hospital[Title] OR practices[Title] OR practice[Title] OR provider[Title] OR providers[Title])

EPR (n=0)
(((EPR[Title] OR "electronic patient record"[Title] OR "electronic patient records"[Title])) AND (cost[Title] OR costs[Title] OR revenue[Title] OR benefit[Title] OR return[Title] OR ROI[Title] OR value[Title] OR efficiency[Title] OR productivity[Title])) AND (hospitals[Title] OR hospital[Title] OR practices[Title] OR practice[Title] OR provider[Title] OR providers[Title])

Analytics (n=10)
(((analytics[Title])) AND (cost[Title] OR costs[Title] OR revenue[Title] OR benefit[Title] OR return[Title] OR ROI[Title] OR value[Title] OR efficiency[Title] OR productivity[Title])) AND (hospitals[Title] OR hospital[Title] OR practices[Title] OR practice[Title] OR provider[Title] OR providers[Title])

AI & Big Data (n=1)
(((AI[Title] OR "artificial intelligence"[Title] OR "big data"[Title])) AND (cost[Title] OR costs[Title] OR revenue[Title] OR benefit[Title] OR return[Title] OR ROI[Title] OR value[Title] OR efficiency[Title] OR productivity[Title])) AND (hospitals[Title] OR hospital[Title] OR practices[Title] OR practice[Title] OR provider[Title] OR providers[Title])

Algorithms, ML & DL (n=17)
(((algorithm[Title] OR algorithms[Title] OR "machine learning"[Title] OR "deep learning"[Title])) AND (cost[Title] OR costs[Title] OR revenue[Title] OR benefit[Title] OR return[Title] OR ROI[Title] OR value[Title] OR efficiency[Title] OR productivity[Title])) AND (hospitals[Title] OR hospital[Title] OR practices[Title] OR practice[Title] OR provider[Title] OR providers[Title])

Decision Support (n=7)
((("decision support"[Title])) AND (cost[Title] OR costs[Title] OR revenue[Title] OR benefit[Title] OR return[Title] OR ROI[Title] OR value[Title] OR efficiency[Title] OR productivity[Title])) AND (hospitals[Title] OR hospital[Title] OR practices[Title] OR practice[Title] OR provider[Title] OR providers[Title])

NLP (n=0)
((("natural language processing"[Title] OR NLP [Title])) AND (cost[Title] OR costs[Title] OR revenue[Title] OR benefit[Title] OR return[Title] OR ROI[Title] OR value[Title] OR efficiency[Title] OR productivity[Title])) AND (hospitals[Title] OR hospital[Title] OR practices[Title] OR practice[Title] OR provider[Title] OR providers[Title])

Telemedicine (n=0)
(((telemedicine[Title])) AND (cost[Title] OR costs[Title] OR revenue[Title] OR benefit[Title] OR return[Title] OR ROI[Title] OR value[Title] OR efficiency[Title] OR productivity[Title])) AND (hospitals[Title] OR hospital[Title] OR practices[Title] OR practice[Title] OR provider[Title] OR providers[Title])

Σ n=79

Σ Σ n=244 (incl. duplicates)
