# Supplementary material for: Economic Value of Data and Analytics for Health Care Providers: Hermeneutic Systematic Literature Review
Source: J Med Internet Res. 2020 Nov 18;22(11):e23315. doi: 10.2196/23315 (PMC7710451; doi:10.2196/23315)
Supplement: Multimedia Appendix 3 [file jmir_v22i11e23315_app3.pdf]

Multimedia Appendix 3: Overview of all published articles screened by title, abstract & text (incl. reasons for exclusion)

Authors & Year	Title	Journal	Included?	Reason for exclusion
**Adler-Milstein J, Green CE, Bates DW (2013)**	A survey analysis suggests that electronic health records will yield revenue gains for some practices and losses for many	Health Affairs	✓	
**Adler-Milstein J, Salzberg C, Franz C, Orav EJ, Newhouse JP, Bates DW (2013)**	Effect of electronic health records on health care costs: longitudinal comparative evidence from community practices	Annals of Internal Medicine	✕	Not provider related
**Almeida JP (2016)**	A disruptive Big data approach to leverage the efficiency in management and clinical decision support in a Hospital	Porto Biomedical Journal	✓	
**Andemariam B, Odesina V, Owarish-Gross J, et al. (2014)**	A fast-track emergency department acute sickle cell pain management algorithm results in fewer hospital admissions, decreased length of stay and increased hospital revenue	The Journal of Pain	✕	No data or analytics (analogue)
**Aponte P, Ballard DJ, Becker E, et al. (2011)**	Exploring Financial and Non-Financial Costs and Benefits of Health Information Technology: The Impact of an Ambulatory Electronic Health Record on Financial and Workflow in Primary Care Practices and Costs of Implementation	Grant Final Report	✕	No economic analysis
**Arunambika T, Senthilvadivu P (2019)**	A survey on cost optimization across cloud storage providers: Offline and Online Algorithms	Journal of Analysis and Computation	✕	Not healthcare related
**Bandera C (2017)**	Value-added service providers for mobile education: empirical challenges and analytics	Electronic Commerce Research	✕	Not healthcare related
**Bazzoli F (2016)**	Analytics All-Stars: HDM Clinical Visionary Clive Fields, MD, believes data can help providers cope with value-based care	Health Data Management	✕	No economic analysis

Authors & Year	Title	Journal	Included?	Reason for exclusion
**Beresniak A, Schmidt A, Proeve J, et al. (2016)**	Cost-benefit assessment of using electronic health records data for clinical research versus current practices: Contribution of the Electronic Health Records for Clinical Research (EHR4CR) European Project	Contemporary Clinical Trials	✕	Not provider related
**Berger A, Zhao Q, Murphy B, Leeper NJ, Ting W, Berger JS (2018)**	Patterns of Utilization and Cost of Health Care Among Patients With Chronic Coronary Artery Disease And/Or Peripheral Arterial Disease in Clinical Practice: Analyses of a Large Us Integrated Claims-EMR Database	Circulation	✕	Not provider related
**Beria P, Bertolin A, Grimaldi R (2018)**	Integration between Transport Models and Cost-Benefit Analysis to Support Decision-Making Practices: Two Applications in Northern Italy	Advances in Operations Research	✕	Not healthcare related
**Brooks PB, Fulton ME (2019)**	Demonstrating advanced practice provider value: Implementing a new advanced practice provider billing algorithm	Journal of the American Association of Nurse Practitioners	✕	No data or analytics (analogue)
**Choi J, Lee S, Lee K, Lee J-h (2017)**	Development of a Decision Support System for Estimation of Transportation Cost of 3PL Provider	Korean Management Science Review	✕	Not healthcare related
**Choi JS, Lee WB, Rhee P-L (2013)**	Cost-benefit analysis of electronic medical record system at a tertiary care hospital	Healthcare Informatics Research	✓	
**Chuang T-YA, Yii N, Nyandowe M, Iyer R (2019)**	Examine the impact of the implementation of an electronic medical record system on operating theatre efficiency at a teaching hospital in Australia	International Surgery Journal	✓	
**Cinaroglu S, Baser O (2017)**	Examination of Technical Efficiency of Public Hospital Associations Using Data Envelopment Analysis and Machine Learning Techniques	Ekonomik Yaklasim	✕	No data or analytics (analogue)
**Coatney K (2018)**	Big Data Analytics Capabilities, The Business Value Of Information Technology, And Healthcare Organizations: The Need For Consensus In Evidence-Based Medical Practices	American Journal of Medical Research	✕	No economic analysis

Authors & Year	Title	Journal	Included?	Reason for exclusion
**Collins LB, Ward CW, Boggs B (2019)**	Dysphagia Severity and Decision Making Algorithm Impact on Length of Hospital Stay, Restraint Use and Cost in Stroke Patients	Stroke	✓	
**Cook J (2016)**	Driving hospital efficiency: Data analytics is the key to insightful asset management	Health Management Technology	✕	No economic analysis
**Cornide-Reyes H, Noël R, Riquelme F, et al. (2019)**	Introducing Low-Cost Sensors into the Classroom Settings: Improving the Assessment in Agile Practices with Multimodal Learning Analytics	Sensors	✕	Not healthcare related
**Cykert S, Lefebvre A (2011)**	Regional extension coordinators: use of practice support and electronic health records to improve quality and efficiency	North Carolina Medical Journal	✕	No economic analysis
**Dad K, Khan MJ, Jie W, Lee MC (2017)**	A low cost genetic algorithm based control scheme for wheelchair control in hospital environment	Journal of Robotics, Networking and Artificial Life	✕	No data or analytics (analogue)
**Dandu N, Zmistowski B, Chen AF, Chapman T, Howley M (2019)**	How are Electronic Health Records Associated with Provider Productivity and Billing in Orthopaedic Surgery?	Clinical Orthopaedics and Related Research	✓	
**De Leon S, Connelly-Flores A, Mostashari F, Shih SC (2010)**	The business end of health information technology: Can a fully integrated electronic health record increase provider productivity in a large community practice?	Journal of Medical Practice Management	✓	
**Demleitner NV (2015)**	How to Change the Philosophy and Practice of Probation and Supervised Release: Data Analytics, Cost Control, Focus on Reentry, and a Clear Mission	Federal Sentencing Reporter	✕	Not healthcare related
**Dias MM, Moreno AS, Maia LSM, et al. (2020)**	A cost-effective algorithm for diagnosis of hereditary angioedema with normal C1 inhibitor: Applying molecular approach to clinical practice	Journal of Allergy and Clinical Immunology: In Practice	✕	No data or analytics (analogue)

Authors & Year	Title	Journal	Included?	Reason for exclusion
**Doub T (2013)**	Your future as a provider: it's all in the data: problem: behavioral health providers lack the analytics capability to extract data's value	Behavioral Healthcare	✕	No economic analysis
**Dulac JD, Pryor RW, Morrissey WW (2017)**	A data-driven approach to improving clinical care and reducing costs the experiences of an academic health center and a hospital system in applying data analytics provide valuable lessons for other organizations that are just beginning such efforts, which are critically important for success under value-based payment	Healthcare Financial Management	✓	
**Elkin PL, Liebow M, Bauer BA, et al. (2010)**	The introduction of a diagnostic decision support system (DXplain™) into the workflow of a teaching hospital service can decrease the cost of service for diagnostically challenging Diagnostic Related Groups (DRGs)	International Journal of Medical Informatics	✓	
**Encinosa WE, Bae J (2013)**	Will meaningful use electronic medical records reduce hospital costs?	The Amercian Journal of Managed Care	✓	
**Everett BR, Sitton JT, Wilson M (2017)**	Efficacy and cost-benefit analysis of a global environmental cleaning algorithm on hospital-acquired infection rates	Journal of Patient Safety	✕	No data or analytics (analogue)
**Fleddermann A, Jones S, James S, Kennedy KF, Main ML, Austin BA (2018)**	Implementation of best practice alert in an electronic medical record to limit lower-value inpatient echocardiograms	The American Journal of Cardiology	✓	
**Fleming NS, Culler SD, McCorkle R, Becker ER, Ballard DJ (2011)**	The financial and nonfinancial costs of implementing electronic health records in primary care practices	Health Affairs	✕	Not provider related
**Furukawa MF (2011)**	Electronic medical records and the efficiency of hospital emergency departments	Medical Care Research and Review	✓	

Authors & Year	Title	Journal	Included?	Reason for exclusion
**Furukawa MF, Raghu T, Shao BB (2010)**	Electronic medical records and cost efficiency in hospital medical-surgical units	INQUIRY: The Journal of Health Care Organization, Provision, and Financing	✓	
**Gardner E (2009)**	Trial runners: A two-physician practice goes on a roll with its EHR, generating extra revenue from clinical trials	Health Data Management	✕	No economic analysis
**Gillespie G (2016)**	IT solves the payment puzzle: Providers deploy analytics, cost estimators as model changes	Health Data Management	✕	No economic analysis
**González-Del Vecchio M, Catalán P, de Egea V, et al. (2015)**	An algorithm to diagnose influenza infection: evaluating the clinical importance and impact on hospital costs of screening with rapid antigen detection tests	European Journal of Clinical Microbiology & Infectious Diseases	✕	No data or analytics (analogue)
**Haq SM (2009)**	Anemia analyzer: algorithm and reflex testing in clinical practice leading to efficiency and cost savings	Studies in Health Technology and Informatics	✕	No data or analytics (analogue)
**Hargreaves JS (2010)**	Will electronic personal health records benefit providers and patients in rural America?	Telemedicine and e-Health	✕	No economic analysis
**Haynes N, Egan D (2017)**	Revisiting the relevance of economic theory to hotel revenue management education and practice in the era of Big Data	Research in Hospitality Management	✕	Not healthcare related
**Highfill T (2019)**	Do hospitals with electronic health records have lower costs? A systematic review and meta-analysis	International Journal of Healthcare Management	✓	
**Hill Jr RG, Sears LM, Melanson SW (2013)**	4000 clicks: a productivity analysis of electronic medical records in a community hospital ED	The American Journal of Emergency Medicine	✕	No economic analysis

Authors & Year	Title	Journal	Included?	Reason for exclusion
**Hollenbeck SM, Bomar JD, Wenger DR, Yaszay B (2017)**	Electronic medical record adoption: the effect on efficiency, completeness, and accuracy in an academic orthopaedic practice	Journal of Pediatric Orthopaedics	✓	
**Huang L, Shea AL, Qian H, Masurkar A, Deng H, Liu D (2019)**	Patient clustering improves efficiency of federated machine learning to predict mortality and hospital stay time using distributed electronic medical records	Journal of Biomedical Informatics	✕	No economic analysis
**Huerta TR, Thompson MA, Ford EW, Ford WF (2013)**	Electronic health record implementation and hospitals' total factor productivity	Decision Support Systems	✓	
**Hughes CA, Guirguis LM, Wong T, Ng K, Ing L, Fisher K (2011)**	Influence of pharmacy practice on community pharmacists' integration of medication and lab value information from electronic health records	Journal of the American Pharmacists Association	✕	No economic analysis
**James T, Jones C, Lafrance D, Nix M (2014)**	Applying Data Analytics to Value-Based Cancer Care: Effects and Cost of Hospital Reencounters Following Cancer Surgery	Value in Health	✕	Not provider related
**Jang Y, Lortie MA, Sanche S (2014)**	Return on investment in electronic health records in primary care practices: a mixed-methods study	JMIR Medical Informatics	✓	
**Jones KM, Afnan T (2019)**	"For the benefit of all students": Student trust in higher education learning analytics practices	Proceedings of the Association for Information Science and Technology	✕	Not healthcare related
**Joseph M (2010)**	Meaningful streamlining: Hybrid practice management/EMR system boosts quality, reduces costs	Modern Healthcare	✓	
**Kadish SS, Mayer EL, Jackman DM, et al. (2018)**	Implementation to Optimization: A Tailored, Data-Driven Approach to Improve Provider Efficiency and Confidence in Use of the Electronic Medical Record	Journal of Oncology Practice	✓	

Authors & Year	Title	Journal	Included?	Reason for exclusion
**Kaneko K, Onozuka D, Shibuta H, Hagihara A (2018)**	Impact of electronic medical records (EMRs) on hospital productivity in Japan	International Journal of Medical Informatics	✓	
**Kazley AS, Ozcan YA (2009)**	Electronic medical record use and efficiency: A DEA and windows analysis of hospitals	Socio-Economic Planning Sciences	✓	
**Keenan MM, Firchau M, Kimura R, Fogg L, Rousseau J (2018)**	Customizing Functionality in an Electronic Health Record to Capture Value of Provider-Specific Services	Cin: Computers, Informatics, Nursing	✗	No economic analysis
**Keller ME, Kelling SE, Cornelius DC, Oni HA, Bright DR (2015)**	Enhancing practice efficiency and patient care by sharing electronic health records	Perspectives in Health Information Management	✗	Not provider related
**Kelley M, Padula W (2019)**	Development of a Machine Learning Algorithm to Predict Future Risk of Patients for high-cost hospital-acquired pressure injuries	Value in Health	✗	No economic analysis
**Kosiorek D (2014)**	Analyze cost, usability features carefully when considering EHR switch: Use the experience your practice gained during its first EHR implementation to make your new system work for you	Medical Economics	✗	No economic analysis
**Kramer H, Lin G, Curtin C, Crowe E, Granderson J (2019)**	Building analytics and monitoring-based commissioning: industry practice, costs, and savings	Energy Efficiency	✗	Not healthcare related
**Lal LS, Raju A, Miller L-A, Chen H, Arbuckle R, Sansgiry SS (2011)**	Impact of changes in reimbursement policies and institutional practice algorithm for utilization of erythropoietic-stimulating agents on treatment patterns and costs in anemic lymphoma patients	Supportive Care in Cancer	✗	Not provider related
**Lee YH (2018)**	Efficiency improvement in a busy radiology practice: determination of musculoskeletal magnetic resonance imaging protocol using deep-learning convolutional neural networks	Journal of Digital Imaging	✓	

Authors & Year	Title	Journal	Included?	Reason for exclusion
**Li K, Naganawa S, Wang K, et al. (2012)**	Study of the cost-benefit analysis of electronic medical record systems in general hospital in China	Journal of Medical Systems	✓	
**Mayeda E, Gerland A (2018)**	Using analytics to design provider networks for value-based contracts: To build a successful provider network in a value-based world, healthcare organizations should collect and analyze several key pieces of data	Healthcare Financial Management	✗	No economic analysis
**McFarland A (2017)**	A cost utility analysis of the clinical algorithm for nasogastric tube placement confirmation in adult hospital patients	Journal of Advanced Nursing	✗	No data or analytics (analogue)
**Mercer C, Bell G, Low N, Estcourt C, Brook G, Cassell J (2012)**	Quantifying the public health value of provider-led partner notification using an evidence-based algorithm with routinely-collected data	Sexually Transmitted Infections	✗	No data or analytics (analogue)
**Mertz L (2014)**	Saving Lives and Money with Smarter Hospitals: Streaming analytics, other new tech help to balance costs and benefits	IEEE pulse	✗	Not provider related
**Mertz L (2018)**	Machine Learning Takes on Health Care: Leonard D'Avolio's Cyft Employs Big Data to Benefit Patients and Providers	IEEE pulse	✗	No economic analysis
**Morrissey J (2015)**	Re-imaging imaging: Providers looking to analytics tools to cope with value-based care	Health Data Management	✗	No economic analysis
**O'Reilly-Shah V, Easton G, Gillespie S (2017)**	Assessing the global reach and value of a provider-facing healthcare app using large-scale analytics	BMJ Global Health	✗	No economic analysis
**Ogundipe OF, Van den Bergh R, Thierry B, et al. (2019)**	Better care for babies: the added value of a modified reverse syphilis testing algorithm for the treatment of congenital syphilis in a maternity Hospital in Central African Republic	BMC pediatrics	✗	No data or analytics (analogue)

Authors & Year	Title	Journal	Included?	Reason for exclusion
**Okumura LM, Veroneze I, Bugardt CI, Fragoso MF (2016)**	Effects of a computerized provider order entry and a clinical decision support system to improve cefazolin use in surgical prophylaxis: a cost saving analysis	Pharmacy Practice (Granada)	✓	
**Patt DA, Nubie M, Kazzaz DR, et al. (2012)**	Clinical decision support tools to improve quality and practice efficiency across a large network of oncology practices	Journal of Clinical Oncology	✕	No data or analytics (analogue)
**Peck JS, Benneyan JC, Nightingale DJ, Gaehde SA (2014)**	Characterizing the value of predictive analytics in facilitating hospital patient flow	IIE Transactions on Healthcare Systems Engineering	✓	
**Pyron L, Carter-Templeton H (2019)**	Improved Patient Flow and Provider Efficiency After the Implementation of an Electronic Health Record	CIN: Computers, Informatics, Nursing	✓	
**Qinglan Y, Yushan Z (2015)**	The value and practice of learning analytics in computer assisted language learning	Studies in Literature and Language	✕	Not healthcare related
**Quadros DG, Neville IS, Urena FM, Solla DJF, Paiva WS, Teixeira M (2019)**	Safety and Costs Analysis of a Fast-track Algorithm for Early Hospital Discharge After Brain Tumor Surgery	Neurosurgery	✓	
**Qutub M, Govindan P, Vattappillil A (2019)**	Effectiveness of a Two-Step Testing Algorithm for Reliable and Cost-Effective Detection of Clostridium difficile Infection in a Tertiary Care Hospital in Saudi Arabia	Medical Sciences	✕	No data or analytics (analogue)
**Rahmawati SR (2019)**	Could We Derive Benefit From Implementing Electronic Medical Records In Hospital?: A Structured Evidence and Narrative Review	ARKESMAS (Arsip Kesehatan Masyarakat)	✓	
**Redd TK, Read-Brown S, Choi D, Yackel TR, Tu DC, Chiang MF (2014)**	Electronic health record impact on productivity and efficiency in an academic pediatric ophthalmology practice	Journal of American Association for Pediatric Ophthalmology and Strabismus	✓	

Authors & Year	Title	Journal	Included?	Reason for exclusion
**Reis ZSN, Maia TA, Marcolino MS, Becerra-Posada F, Novillo-Ortiz D, Ribeiro ALP (2017)**	Is there evidence of cost benefits of electronic medical records, standards, or interoperability in hospital information systems? Overview of systematic reviews	JMIR Medical Informatics	✓	
**Rivera J, Delaney S (2015)**	Using business analytics to improve outcomes: Orlando Health is taking advantage of customized solutions to get its hospital and physician practice revenue cycle systems in synch and strengthen its organization operationally and financially	Healthcare Financial Management	✓	
**Rommers MK, Zwaveling J, Guchelaar H-J, Teepe-Twiss IM (2013)**	Evaluation of rule effectiveness and positive predictive value of clinical rules in a Dutch clinical decision support system in daily hospital pharmacy practice	Artificial Intelligence in Medicine	✕	No data or analytics (analogue)
**Rosenbluth G, Wilson SD, Maselli JH, Auerbach AD (2011)**	Analgesic prescribing practices can be improved by low-cost point-of-care decision support	Journal of Pain and Symptom Management	✕	No data or analytics (analogue)
**Samir M (2009)**	Anemia analyzer: algorithm and reflex testing in clinical practice leading to efficiency and cost savings	Advances in Information Technology and Communication in Health	✕	No data or analytics (analogue)
**Schnaus MJ, Michalik M, Skarda P (2017)**	Effects of Electronic Medical Record Display on Provider Ordering Behavior: Leveraging the EMR to Improve Quality and Costs	The American Journal of Medicine	✓	
**Scholz J, Portela LD, Abe TMO, et al. (2016)**	Cost-effectiveness analysis of smoking-cessation treatment using electronic medical records in a cardiovascular hospital	Clinical Trials and Regulatory Science in Cardiology	✕	No data or analytics (analogue)
**Schouten P (2013)**	Big data in health care: solving provider revenue leakage with advanced analytics	Healthcare Financial Management	✓	

Authors & Year	Title	Journal	Included?	Reason for exclusion
**Séroussi B, Soulet A, Spano J-P, et al. (2013)**	Which patients may benefit from the use of a decision support system to improve compliance of physician decisions with clinical practice guidelines: a case study with breast cancer involving data mining	Studies in Health Technology and Informatics	✕	No economic analysis
**Silow-Carroll S, Edwards JN, Rodin D (2012)**	Using electronic health records to improve quality and efficiency: the experiences of leading hospitals	Issue Brief (Commonw Fund)	✓	
**Stekel SF, Long Z, Tradup DJ, Hangiandreou NJ (2019)**	Use of Image-Based Analytics for Ultrasound Practice Management and Efficiency Improvement	Journal of Digital Imaging	✓	
**Stoves J, Connolly J, Cheung CK, et al. (2010)**	Electronic consultation as an alternative to hospital referral for patients with chronic kidney disease: a novel application for networked electronic health records to improve the accessibility and efficiency of healthcare	Quality and Safety in Health Care	✓	
**Taghavi A, Monem H (2018)**	Increasing the Efficiency of Using CCU Beds of Hospitals through Optimization and Combination of Genetic Algorithm and Imperialist Competitive	Journal of Health and Biomedical Informatics	✕	No economic analysis
**Terry K (2011)**	Rev up your EHR: how to optimize performance; Learn ways to increase revenue, improve practice efficiency and quality	Medical Economics	✕	No economic analysis
**Terry K (2014)**	Value-based incentives can help practices offset EHR costs	Medical Economics	✓	
**Terry K (2014)**	Use your EHR system to boost practice revenue	Contemporary OB/GYN	✕	No economic analysis
**Teuben M, Löhr N, Jensen KO, et al. (2019)**	Improved pre-hospital care efficiency due to the implementation of pre-hospital trauma life support (PHTLS®) algorithms	European Journal of Trauma and Emergency Surgery	✕	No data or analytics (analogue)

Authors & Year	Title	Journal	Included?	Reason for exclusion
**Teufel II RJ, Kazley AS, Ebeling MD, Basco Jr WT (2012)**	Hospital electronic medical record use and cost of inpatient pediatric care	Academic Pediatrics	✕	No economic analysis
**Thompson G, O'Horo JC, Pickering BW, Herasevich V (2015)**	Impact of the Electronic Medical Record on Mortality, Length of Stay, and Cost in the Hospital and ICU: A Systematic Review and Metaanalysis	Critical Care Medicine	✓	
**Urbinati A, Bogers M, Chiesa V, Frattini F (2018)**	How do Provider Companies Create and Capture Value from Big Data Technology: An Exploratory Multiple Case Study Analysis	Academy of Management Global Proceedings	✕	Not healthcare related
**Urbinati A, Bogers M, Chiesa V, Frattini F (2019)**	Creating and capturing value from Big Data: A multiple-case study analysis of provider companies	Technovation	✕	Not healthcare related
**Uslu A, Stausberg J (2011)**	Value of the electronic medical record for hospital care: A review of the literature	Journal of Healthcare Engineering	✓	
**Van der Maas M, Steuten L (2015)**	A PCT-algorithm to guide antibiotic therapy in patients hospitalized with COPD exacerbations leads to net cost savings by reducing frequency and duration of antibiotic use as compared to current practice	Value in Health	✕	No data or analytics (analogue)
**Verhoye E, Vandecandelaere P, De Beenhouwer H, et al. (2015)**	A hospital-level cost-effectiveness analysis model for toxigenic Clostridium difficile detection algorithms	Journal of Hospital Infection	✕	No data or analytics (analogue)
**Voermans AM, Mewes JC, Broyles MR, Steuten LM (2019)**	Cost-Effectiveness Analysis of a Procalcitonin-Guided Decision Algorithm for Antibiotic Stewardship Using Real-World US Hospital Data	Omics: A Journal of Integrative Biology	✕	No data or analytics (analogue)
**Voleti VB, Braunstein AL, Mahabir C, Schrier A, Chiang MF (2011)**	Evaluation Of An Academic Eye Clinic'S Practice Efficiency During The Early Transition Period To An Electronic Health Record System	Investigative Ophthalmology & Visual Science	✓	

Authors & Year	Title	Journal	Included?	Reason for exclusion
**Wagholikar KB, Hankey RA, Decker LK, et al. (2015)**	Evaluation of the effect of decision support on the efficiency of primary care providers in the outpatient practice	Journal of Primary Care & Community Health	✓	
**Wang H, Cui Z, Chen Y, Avidan M, Abdallah AB, Kronzer A (2018)**	Predicting hospital readmission via cost-sensitive deep learning	IEEE/ACM Transactions on Computational Biology and bioinformatics	✓	
**Wilson G, Kishk M (2013)**	A decision support model proving 'value for money' selection of elements and components on hospital refurbishments: development of a functioning prototype	Construction Engineering	✕	Not healthcare related
**Wuest TK (2019)**	CORR Insights®: How are Electronic Health Records Associated with Provider Productivity and Billing in Orthopaedic Surgery?	Clinical Orthopaedics and Related Research	✕	No economic analysis
**Xiao Y, Meng B, Tang T, Wu Y, Xie R (2017)**	Research and Practice for Leveraging Big Data in Smart Education: Value and Path	e-Education Research	✕	Not healthcare related
**Zeng Y-c, He T, Li X-h, Li J-q, Xiao F (2012)**	Study on the Cost Audit of Electronic Medical Records of 65855 Patients Discharged from the Hospital	Hospital Administration Journal of Chinese People's Liberation Army	✕	No economic analysis
**Zhang J, Zheng T, Fang J (2013)**	Application Prospect of EPR System in Total Cost Accounting of Hospitals	Chinese Health Economics	✕	No economic analysis
**Zimlichman E, Keohane C, Franz C, et al. (2013)**	Return on investment for vendor computerized physician order entry in four community hospitals: the importance of decision support	The Joint Commission Journal on Quality and Patient Safety	✓	
