# Supplementary material for: Economic Value of Data and Analytics for Health Care Providers: Hermeneutic Systematic Literature Review
Source: J Med Internet Res. 2020 Nov 18;22(11):e23315. doi: 10.2196/23315 (PMC7710451; doi:10.2196/23315)
Supplement: Multimedia Appendix 4 [file jmir_v22i11e23315_app4.pdf]

Multimedia Appendix 4: Syntheses of included studies

Study	Objective	Findings	Methodology & data	Region	Categorization
		**Systematic literature search (Google Scholar & PubMed, n=43)**			
**[20] Highfill, T. (2019): Do hospitals with electronic health records have lower costs? A systematic review and meta-analysis**	→ Presents a systematic review and meta-analysis of literature examining the effect of EHR introduction on hospital costs thereby differentiating between “basic” (incl. data repository & information system) and “advanced” (incl. computerized physician order entry & clinical decision support) EHRs	→ Overall, the meta-analysis revealed 1.1% to 13.8% lower costs for hospitals that deployed an EHR (95% CI)<br>→ Hospitals with “basic” EHRs were found to have 0.3% to 23.8% lower costs (95% CI)<br>→ Hospitals with “advanced” EHRs showed 3% lower costs, however this effect was not statistically significant	Systematic literature review and meta-analysis based on 7 articles with no limitation on publication date	Various	→ Electronic Health Records<br>→ Direct positive economic impact
**[21] Rahmawati et al. (2018): Could We Derive Benefit From Implementing Electronic Medical Records In Hospital?: A Structured Evidence and Narrative Review**	→ Narrative review of the impact of EHR introductions on efficiency and outcomes	→ Overall, the impact of EHR introductions on provider efficiency extracted from the included studies was ambiguous<br>→ The authors identified several benefits of EHR introduction including reduced length of stay (LOS) or decreased infection rate<br>→ However, studies were partly contradictory especially related to the impact on average LOS with some studies showing increased times<br>→ Identified disadvantages included increased costs per patient accompanied by lower efficiency in medical-surgical settings	Narrative review including 12 studies from 2008 to 2018 in a qualitative synthesis in accordance with the preferred reporting items for systematic reviews and meta-analyses (PRISMA) statement	Various	→ Electronic Health Records<br>→ Mixed economic impact
**[22] Reis et al. (2017): Is there evidence of cost benefits of electronic medical records, standards, or interoperability in hospital information systems?**	→ Presents an overview of systematic reviews with the primary goal of evaluating impact of EHR, interoperability and standards on cost-effectiveness, as well as,	→ The authors identified preliminary benefits of EHRs and related components on the quality of care<br>→ Overall, cost-effectiveness could not clearly be examined due to heterogeneous study designs, but mixed effects were identified for	Overview of systematic reviews through February 2016 including 6 studies in accordance with the preferred reporting items for systematic reviews and meta-analyses (PRISMA) statement	Various	→ Electronic Health Records<br>→ Mixed economic impact

Study	Objective	Findings	Methodology & data	Region	Categorization
**Overview of systematic reviews**	quality of care as secondary goal	EHRs without Health Information Exchange (HIE)			
**[23] Uslu et al. (2011): Value of the electronic medical record for hospital care: A review of the literature**	→ Presents a literature review on the impact of EHR on quality of care and hospital costs	→ 5 studies provided slightly positive indices regarding a positive effect on care outcomes after EHR introduction and 1 found mixed results<br>→ The authors stated that 5 studies also provided evidence for a positive impact on cost for care providers mainly linked to time savings required for administrative and archiving work, as well as, savings in nursing costs<br>→ 1 study implied negative effects on provider economics<br>→ Nevertheless, many of the mentioned studies did not provide actual costs savings, but rather efficiency improvements that could potentially lead to savings	Literature review based on 7 included studies in the field of interest resulting from a search between 2004 and 2010	Various	→ Electronic Health Records<br>→ Mixed economic impact
**[30] Thompson et al. (2015): Impact of the Electronic Medical Record on Mortality, Length of Stay, and Cost in the Hospital and ICU: A Systematic Review and Metaanalysis**	→ Presents a systematic review and meta-analysis of the effects of health information technology (HIT) on mortality, length of stay (LOS) and costs for hospitals	→ Overall, electronic interventions did not show a substantial effect on mortality, LOS or costs<br>→ The only statistically significant effect was the positive impact of surveillance systems on mortality, other effects on quality indicators were not statistically significant<br>→ Cost effects were considered by 14 studies, however, could not be quantitatively synthesized due to significant heterogeneity, however, 8 of the 14 studies showed a decrease in cost after an electronic intervention	Systematics review and meta-analysis based on 45 studies through July 2013 in the field of interest of which 26 were included in the quantitative meta-analysis deploying Dersimonian and Laird random effects models	Various	→ Electronic Health Records<br>→ Mixed economic impact
**[31] Choi et al. (2013): Cost-benefit analysis of electronic medical record system at a tertiary care hospital**	→ Examines a cost-benefit analysis (CBA) for an EHR implementation at the Samsung Medical Center general hospital	→ Negative 5-year net-present-value (NPV) of $385,000 and a positive 8-year NPV of $3,617,000 were determined<br>→ The positive 8-year NPV was achieved by fully cutting "legacy" costs like paper records (incl. related FTEs) and by generating	Cost-benefit analysis (CBA) based on retrospective financial data of the Samsung Medical Center general hospital and expert interviews to allocate the share of benefits and cost	South Korea	→ Electronic Health Records<br>→ Mixed economic impact

Study	Objective	Findings	Methodology & data	Region	Categorization
		additional revenue through medical transcriptions and by repurposing former paper record space into clinical space	reductions accredited to EHR introduction		
**[32] Li et al. (2012): Study of the cost-benefit analysis of electronic medical record systems in general hospital in China**	→ Examines the 6-year return on investment (ROI) of an EHR implementation in a Chinese general hospital	→ The EHR installation broke even after 3 years and showed a positive 6-year ROI of $559,025<br>→ The positive ROI was mainly driven by savings in new record creation, FTE savings, savings related to ADEs, improved charge capture and decreased billing errors	Return on investment calculation based on financial data and expert opinions on potential savings at a 700-bed general hospital with benefits/saving categories including storage requirements, record maintenance, new record creation, charge capture, adverse drug effects (ADE) and others	China	→ Electronic Health Records<br>→ Direct positive economic impact
**[33] Zimlichman et al. (2013): Return on investment for vendor computerized physician order entry in four community hospitals: the importance of decision support**	→ Examines the return on investment (ROI) of a vendor computerized physician order entry (CPOE) on the basis of a reduction in preventable adverse drug effects (ADE)	→ Hospital group A (2 hospitals) had a comparably lower installation cost for the vendor CPOE and was able to break even after 8 years and achieved a 11.3% 10-year ROI<br>→ Hospital group B (2 hospitals) with comparably higher installation costs showed a negative 10-year ROI of 3.1%<br>→ The authors attributed the rather low ROIs and a long break-even timeline for group A to a lack of clinical decision support tools at all hospitals	10-year cost-benefit analysis including ROI, net cash flow and breakeven points evaluating the introduction of a CPOE at four hospitals with benefits being only based on reductions in ADEs	USA	→ Electronic Health Records<br>→ Mixed economic impact
**[34] Jang et al. (2014): Return on investment in electronic health records in primary care practices: a mixed-methods study**	→ Examines the return on investment (ROI) of EHR introduction for 17 Canadian primary care clinics	→ Primary care clinics typically recovered their EHR investment within 6.2 to 17.4 months (95% CI) going along with increased patient volume and practice revenue<br>→ More recent EHR systems and those using flow sheets showed shorter break-even timelines<br>→ Surveyed physicians stated benefits such as better ability to manage results, faster information retrieval and better patient encounter preparation	Mixed-methods study determining break-even times based on clinic financial and care data, as well as, physician surveys at 132 community-based, primary care clinics in Canada	Canada	→ Electronic Health Records<br>→ Direct positive economic impact

Study	Objective	Findings	Methodology & data	Region	Categorization
**[35] Adler-Milstein et al. (2013): A survey analysis suggests that electronic health records will yield revenue gains for some practices and losses for many**	→ Examines the 5-year return-on-investment (ROI) of EHR implementation for a network of 49 community practices	→ EHR introduction had a negative 5-year ROI of $43,743 per physician on average<br>→ 27% of practices achieved a positive ROI by fully cutting “legacy” costs like paper records and dictation and also leveraging EHR for revenue generation via increased efficiency and billing	Cost-benefit analysis (CBA) based on retrospective practice accounting data and a survey completed by practices allocating the share of benefits and cost reductions accredited to EHR introduction	United States of America (USA)	→ Electronic Health Records<br>→ Direct negative economic impact
**[36] Encinosa et al. (2013): Will meaningful use electronic medical records reduce hospital costs?**	→ Examines the impact of establishing different levels of EHR (Meaningful Use functions 1-5) on Adverse-Drug-Effects and respective costs	→ Adopting all 5 functions reduced ADEs from 3.6% to 1.4% with the potential to save the associated costs of $4,790 per avoided ADE ($p<0.01$)	Multivariate logit regression analysis based on Florida inpatient hospitalization data, American Hospital Association Annual Survey data and Medicare Hospital Compare data	USA	→ Electronic Health Records<br>→ Direct positive economic impact
**[37] Joseph, M. (2010): Meaningful streamlining: Hybrid practice management/EMR system boosts quality, reduces costs**	→ Presents personnel cost savings related to an EHR introduction for the US-based Westmed Medical Group provider network	→ The introduction of an EHR reduced the number of paper record FTEs by 17 FTEs, saving more than $6 million in 5 years	Qualitative case study of the Westmed Medical Group network	USA	→ Electronic Health Records<br>→ Direct positive economic impact
**[39] Silow-Carroll et al. (2012): Using electronic health records to improve quality and efficiency: the experiences of leading hospitals**	→ Presents the experiences of 9 hospitals that implemented an EHR in terms of quality of care and efficiency	→ Time efficiency: Mostly anecdotal information that revealed an ambiguous effect of EHRs on time savings for physicians, however, one hospital reported it was able to reduce or redirect to higher-value activities 190 FTEs since the EHR took over certain tasks<br>→ Patient volume: Hospitals reported a positive effect on “throughput”, e.g., the time to assign a bed for a newly admitted patient decreased by 90 minutes at one hospital and by 80% when admitting an emergency department patient<br>→ Redundancies: It was perceived by all hospitals that redundant tests were reduced with one hospital reporting a laboratory test reduction of 18% and a radiology examination decrease of 6.3%	Qualitative and semi-quantitative case studies of 9 major US-based hospitals (Carilion Roanoke Memorial Hospital, Virginia; Doctors Hospital, Ohio; Geisinger Wyoming Valley Hospital, Pennsylvania; Gundersen Lutheran Medical Center, Wisconsin; Metro Health Hospital, Michigan; New York-Presbyterian Hospital, New York; Sentara Norfolk General Hospital, Virginia; VA Central Iowa Health Care System, Iowa; Yale-New Haven Hospital, Connecticut)	USA	→ Electronic Health Records<br>→ Direct positive economic impact

Study	Objective	Findings	Methodology & data	Region	Categorization
		→ Billing: One hospital reported it was able to reduce write-offs for care associated to inadequate documentation of the services or required waivers<br>→ Return on investment (ROI): One hospital calculated the ROI of the EHR estimating an annualized benefit of $50.7 million and breaking even after 5 years			
**[40] Schnaus et al. (2017): Effects of Electronic Medical Record Display on Provider Ordering Behavior: Leveraging the EMR to Improve Quality and Costs**	→ Examines the impact of a temporary change in the preselected type of complete blood count (CBC) test (with vs. without differentials in an EHR's computerized physician order entry (CPOE) tool (i.e. physicians searching for "complete blood count" in the CPOE tool had the CBC with differentials pre-selected for a certain period of time)	→ Significantly more CBC tests with differentials were ordered in the change period vs. in the time before or after the change period<br>→ Of CBCs ordered, 37.7% included differentials before the change period compared to 50% during the change period ($p<0.01$)<br>→ Since costs for a CBC without differentials was $0.80 and $2.92 with differentials, this temporary change in the tool resulted in an average daily cost increase for CBC testing of $293.1<br>→ This change could mainly be attributed to a simple change in pre-selection of the CPOE tool since other effects were controlled for	Linear regression statistical analysis for CBCs ordered at the Regions Hospital in Minnesota in 2016 before (June 16 to July 5), during (July 6 to July 28) and after (July 29 to August 20) the change with sample sizes of 13,242, 14,017 and 13,238 respectively; cost data obtained from internal laboratory operations	USA	→ Electronic Health Records<br>→ Direct negative economic impact
**[41] Terry, K. (2014): Value-based incentives can help practices offset EHR costs**	→ Presents two case studies of how EHR systems can support in generating additional revenue from participating in value-based payment or bonus schemes	→ Not having an EHR is seen to come with the "opportunity costs" of not being able to participate in value-based schemes such as accountable care organizations (ACO), patient-centered medical homes or pay-for-performance programs<br>→ The first case study presents a 7-doctor nephrology practice that is a member of an ACO and participates in the Medicare Shared Savings Program thereby generating additional bonus revenues of an undisclosed number<br>→ The second case study presents an internal medicine practice participates in an ACO and collects pay-for-performance payments, also	Qualitative case studies of a 7-doctor nephrology practice and an internal medicine practice	USA	→ Electronic Health Records<br>→ Direct positive economic impact

Study	Objective	Findings	Methodology & data	Region	Categorization
		the practice generates some revenue from sharing quality data extracted from the EHR with public registries			
**[42] Dandu et al. (2019): How are Electronic Health Records Associated with Provider Productivity and Billing in Orthopaedic Surgery?**	→ Examines the impact of EHR introduction on patient volumes (productivity) and billing in orthopedic surgery	→ EHR introduction did not lead to higher overall volumes, i.e. productivity in general<br>→ Only total knee arthroplasty volumes increased by 4-7 surgeries (95% CI, p<0.001)<br>→ EHR introduction did not affect billing	Retrospective multivariate analysis based on publicly available Medicare Utilization and Payment datasets	USA	→ Electronic Health Records<br>→ Neutral economic impact
**[43] Redd et al. (2014): Electronic health record impact on productivity and efficiency in an academic pediatric ophthalmology practice**	→ Examines the impact of EHR introduction in a pediatric ophthalmology department on productivity in terms of patient volume and efficiency in terms of charting time and after-hours charting	→ A non-statistically significant 11% decrease (p=0.18) in clinical volume after EHR introduction was identified<br>→ Since no data on efficiency before EHR introduction in terms of charting was available, no efficiency impact of EHR could be shown	Retrospective statistical analysis based on patient volume data of four physicians pre- and post- EHR introduction with charting time and after-hours charting being considered only post-introduction	USA	→ Electronic Health Records<br>→ Neutral economic impact
**[44] Voletia et al. (2011): Evaluation Of An Academic Eye Clinic's Practice Efficiency During The Early Transition Period To An Electronic Health Record System**	→ Examines the impact of an EHR introduction on patient volume and visit cycle times at an eye clinic of an academic medical center	→ No statistically significant changes from before to after EHR installation could be observed in patient volume (p=0.31) and cycle times (p=0.92)	Retrospective statistical comparison of monthly patient visits and patient cycle times before and during the first three months after EHR introduction	USA	→ Electronic Health Records<br>→ Neutral economic impact
**[45] Kaneko et al. (2018): Impact of electronic medical records (EMRs) on hospital productivity in Japan**	→ Examines the effect of EHR introduction in terms of labor productivity and multi-factor productivity (MFP) differentiating between "early adopter", "follower" and "late adopter" hospitals	→ EHR implementation had a significant negative impact on MFP growth only for the "late adopters" (0.31-0.82 OR, 95% CI)<br>→ No statistically significant relation was found for labor productivity growth	Logistic regression analysis of labor productivity and MFP growth based on data between 2006 and 2015 from 658 municipal hospitals in Japan	Japan	→ Electronic Health Records<br>→ Indirect negative economic impact
**[46] De Leon et al. (2010): The business end of health information technology. Can a fully integrated electronic**	→ Examines productivity of a 75-physician practice before and after EHR implementation	→ Productivity decreases were observed at the time of EHR implementation	Longitudinal mixed model analysis of the productivity of 75 physicians in terms of patient volume at a large	USA	→ Electronic Health Records<br>→ Indirect positive economic impact

Study	Objective	Findings	Methodology & data	Region	Categorization
**health record increase provider productivity in a large community practice?**		→ Productivity increased by 1.7% per month (p<0.001) per provider after the EHR was fully implemented<br>→ The majority of gains could be associated to a newly introduced pay-for-performance program enabled by data capture of the EHR	urban primary care practice between January 2005 and February 2009 before and after implementing an EHR		
**[47] Kadish et al. (2018): Implementation to Optimization: A Tailored, Data-Driven Approach to Improve Provider Efficiency and Confidence in Use of the Electronic Medical Record**	→ Examines the effect of EHR training for physicians on physician confidence with system and efficiency in terms of time in system (TIS)	→ Physicians reported a significant 36%-point increase in confidence using the EHR system after the training (p<0.001)<br>→ Overall efficiency, i.e. TIS, showed only minimal improvements of less than 1 minute per appointment which was not statistically significant (p=0.1)	Semi-quantitative physician survey analysis to examine physician confidence and statistical comparison of TIS before and after training via paired Wilcoxon test	USA	→ Electronic Health Records<br>→ Neutral economic impact
**[48] Chuang et al. (2019): Examine the impact of the implementation of an electronic medical record system on operating theatre efficiency at a teaching hospital in Australia**	→ Examines the impact on operating theater efficiency after an EHR introduction in terms of operation sessions starting late, average delay time and cancellations	→ The EHR implementation resulted in temporarily reduced efficiency, i.e. an increase in delays in theatre start times from 13.2% to 88.0% of sessions during the first month post implementation (p<0.0001)<br>→ Efficiency improved post implementation and got back to normal efficiency after 4 months of EHR usage (p<0.01)	Retrospective statistical analysis of operating theater data between September 2018 and January 2019 at a major acute teaching hospital	Australia	→ Electronic Health Records<br>→ Indirect negative economic impact
**[49] Furukawa et al. (2010): Electronic medical records and cost efficiency in hospital medical-surgical units**	→ Examines the effect of EHR introduction on the efficiency of medical-surgical units in hospitals	→ EHR implementation was on average associated with lower efficiency in medical-surgical units<br>→ Fully fledged EHR systems incl. computerized physician order entry (CPOE) & clinical decision support (CDS) did not have a significant effect on efficiency	Stochastic frontier analysis (SFA) based on EHR implementation data from the Healthcare Information and Management Systems Society (HIMSS) databases and on costs and nurse staffing from the Annual Financial Disclosure Reports of the California Office of Statewide Health Planning and Development (OSHPD) of 365 Californian hospitals	USA	→ Electronic Health Records<br>→ Indirect negative economic impact
**[50] Hollenbeck et al. (2017): Electronic medical record adoption: the effect on**	→ Examines the impact of EHR introduction in the orthopedic department of	→ No significant difference in monthly patient volumes was identified (p=0.075), however, more physicians were needed per patient	Retrospective comparative study at a 10-surgeon academic medical center	USA	→ Electronic Health Records

Study	Objective	Findings	Methodology & data	Region	Categorization
**efficiency, completeness, and accuracy in an academic orthopaedic practice**	a hospital on efficiency in terms of patient volume, physicians needed per patient and record completeness	resulting in a 19% reduction in volume per physician (p<0.001)<br>→ EHRs were 1.3 times more likely to include all important medical information<br>→ Physician surveys revealed concerns regarding efficiency and increased "off-hours" work for record keeping	based on 60 paper charts, 60 EHRs, billing data and a physician survey to qualitatively assess satisfaction and perceived efficiency		→ Indirect negative economic impact
**[51] Huerta et al. (2012): Electronic health record implementation and hospitals' total factor productivity**	→ Examines the impact of different kinds of EHR introductions (never, incremental, "big-bang", etc.) on hospital efficiency measured as Total Factor Productivity (TFP)	→ All kinds of EHR implementation result in lower efficiency of underlying care processes measured as Technological Change (TC) (p=0.11)<br>→ "Big bang", i.e. the implementation of a fully-fledged EHR in a relatively short time-period approach, performing the worst<br>→ Some approaches were able to reach a slight improvement in overall productivity (TFP), however mainly linked to Technical Efficiency Change (EFFCH) improvements (i.e., physicians worked harder, not smarter)	Frontier analysis measuring hospitals' TFP split into Technical efficiency change (EFFCH) and Technological Change (TC) based on American Hospital Association (AHA) Annual Surveys of Hospitals (2006-2008)	USA	→ Electronic Health Records<br>→ Indirect negative economic impact
**[53] Furukawa et al. (2011): Electronic medical records and the efficiency of hospital emergency departments**	→ Examines the relationship between sophistication of introduced EHR systems (no EHR, minimal EHR, fully functional EHR) and the efficiency of hospital-based emergency departments (ED)	→ Fully functional EHR systems including computerized physician order entry and decision support, compared to no or only basic EHRs, were associated with lower ED length of stay (-11.4% to -33.4%, 95% CI), lower ED wait times (-19.3% to -108.9%, 90% CI) and lower ED treatment time (-7.1% to -19.1%, 95% CI)<br>→ These effects, however, varied significantly by patient acuity level and diagnostic services provided	Survey-weighted ordinary least squares analysis utilizing US National Hospital Ambulatory Medical Care Survey (NHAMCS) data of 35,849 patients from 364 hospital-based EDs	USA	→ Electronic Health Records<br>→ Indirect positive economic impact
**[54] Kazley et al. (2009): Electronic medical record use and efficiency: A DEA and windows analysis of hospitals**	→ Examines the relationship between EHR usage and hospital efficiency and efficiency improvement over time for a sample of 4606 non-federal acute care hospitals in the US	→ A positive relationship between EHR usage and hospital efficiency is observed only for small hospitals (6-100 beds) which are 1.2 to 2.486 times more likely to be more efficient with an EHR (95% CI)<br>→ No efficiency increase over time is associated with EHR usage	Data envelopment analysis for a sample of 4,606 non-federal acute care hospitals determining efficiency and change in efficiency considering non-physician FTEs, beds, capital assets and operating expenses as inputs	USA	→ Electronic Health Records<br>→ Indirect positive economic impact

Study	Objective	Findings	Methodology & data	Region	Categorization
			and admissions and outpatient visits as outputs		
**[55] Pyron et al. (2019): Improved Patient Flow and Provider Efficiency After the Implementation of an Electronic Health Record**	→ Examines the impact of EHR introduction on the efficiency of six urgent care clinics in terms of patient flow and average length of stay (LOS)	→ The EHR introduction had a positive impact on door-to-triage, door-to-provider and door-to-discharge times<br>→ Average LOS decreased from 109 minutes before to 73 minutes after EHR introduction	Retrospective longitudinal study based on several sources of data including an online survey, EHR data, paper chart data & direct observations of physicians at a hospital-owned freestanding urgent care system	USA	→ Electronic Health Records<br>→ Indirect positive economic impact
**[56] Fleddermann et al. (2018): Implementation of best practice alert in an electronic medical record to limit lower-value inpatient echocardiograms**	→ Examines the impact of introducing an automatic alert to avoid unnecessary imaging studies, i.e., transthoracic echocardiography (TTE)	→ Over 209 days, the alert triggered 3,226 times with 20% of the respective TTEs being cancelled, saving the costs for 20% of otherwise performed TTEs	Statistical analysis of TTE orders removed following 3,226 alerts over 209 days at Saint Luke's Mid America Heart Institute	USA	→ Clinical Decision Support<br>→ Direct positive economic impact
**[57] Okumura et al. (2016): Effects of a computerized provider order entry and a clinical decision support system to improve cefazolin use in surgical prophylaxis: a cost saving analysis**	→ Examines the cost savings of implementing a clinical decision support (CDS) algorithm to improve prophylactic cefazolin (antibiotic) usage	→ Following the introduction of a computerized CDS system in 2005 the Defined Daily Doses/100 bed days decreased from 3.3 in 2002 to 2.15 in 2013 with an average yearly decrease of 0.53 (1.26 to -0.2, 95% CI)<br>→ Total cost savings were estimated at around $50,000 between 2005 and 2013	Cross-sectional study based on prophylactic cefazolin usage and costs between 2002 and 2013 determining impact of decision support from 2005 on at a Brazilian university hospital	Brazil	→ Clinical Decision Support<br>→ Direct positive economic impact
**[59] Quadros et al. (2019): Safety and Costs Analysis of a Fast-track Algorithm for Early Hospital Discharge After Brain Tumor Surgery**	→ Examines the impact of introducing a fast-track algorithm to steer decision making on discharges after brain tumor surgery	→ The decision-making algorithm resulted in a significant length of stay reduction of 2 days considering the median ($p<0.001$); without impacting complication or readmission rates<br>→ Significant cost reductions per case where observed post-implementation of $630 per hospitalization ($p=0.043$)	Retrospective cohort study based on data from brain tumor resections from a single neurosurgeon in 2017 comparing statistics pre- and post-implementation	Brazil	→ Clinical Decision Support<br>→ Direct positive economic impact
**[60] Collins et al. (2019): Dysphagia Severity and Decision Making Algorithm Impact on Length of Hospital**	→ Examines the effect of a post-stroke dysphagia (swallowing difficulty) severity and decision-making algorithm on	→ The decision-making algorithm resulted in an earlier insertion of a nasal feeding tube of on average 2.7 days compared to the non-supported regular treatment ($p=0.146$)	Retrospective statistical analysis of data from 40 stroke patients at a US-based hospital	USA	→ Clinical Decision Support<br>→ Direct positive economic impact

Study	Objective	Findings	Methodology & data	Region	Categorization
**Stay, Restraint Use and Cost in Stroke Patients**	length of stay, restraint use and costs	→ Decreases in nasal tube replacements and repeat x-rays translated to decreased costs			
**[61] Wagholikar et al. (2015): Evaluation of the effect of decision support on the efficiency of primary care providers in the outpatient practice**	→ Examines the impact of a clinical decision support (CDS) tool supporting patient chart review, decisions on preventive services and chronic disease management on the time for physicians to generate care recommendations	→ CDS assistance resulted in statistically significant average time savings of 3 minutes 16 seconds (65%) per patient (p<0.0001)	Statistical comparison of time needed for physicians to arrive at a recommendation for patients (tracked via completion time of a checklist) with or without access to a decision support system for 30 patients of a primary care practice	USA	→ Clinical Decision Support<br>→ Indirect positive economic impact
**[62] Elkin et al. (2010): The introduction of a diagnostic decision support system (DXplain™) into the workflow of a teaching hospital service can decrease the cost of service for diagnostically challenging Diagnostic Related Groups (DRGs)**	→ Examines the impact of a diagnostic decision support system on case costs for diagnostically challenging patients in the General Medicine Department of a hospital	→ After tool implementation costs per case for diagnostically challenging patients decreased by between 3.7-19.5% (95% CI)<br>→ The authors hypothesize that the tool aids physicians to consider differential diagnoses potentially guiding them towards the correct diagnosis early on which avoids unnecessary treatments	Retrospective statistical analysis based on financial charges data for 1,173 control group and 564 intervention group cases at a 1,200-bed hospital	USA	→ Clinical Decision Support<br>→ Direct positive economic impact
**[63] Lee, Y. H. (2018): Efficiency improvement in a busy radiology practice: determination of musculoskeletal magnetic resonance imaging protocol using deep-learning convolutional neural networks**	→ Presents the potential utilization of a Deep-Learning Convolutional Neural Network (CNN) to determine Musculoskeletal magnetic resonance imaging (MRI) scanning protocols (i.e., determination of diagnostic performance, image quality, hardware and software and radiologist preferences, etc.)	→ The CNN successfully determined the optimal MRI scanning protocol with a sensitivity of 92.1% and specificity of 95.76% when compared to the radiologist expert opinions<br>→ Nevertheless, the authors only hypothesize and do not prove potential efficiency improvements in practice	Application of a CNN to a test set of 5,258 MRI scans determining one of two overarching optimal MRI protocol based on patient ages, gender, referring departments, examinations and other information	South Korea	→ Advanced Analytics<br>→ Indirect positive economic impact

Study	Objective	Findings	Methodology & data	Region	Categorization
**[65] Wang et al. (2018): Predicting hospital readmission via cost-sensitive deep learning**	→ Presents the utilization of a convolutional neural network (CNN) based on EHR data to predict 30-day, 60-day and 1-year hospital readmissions	→ The developed prediction tool which is also deployed in the hospital achieves a specificity of 89%, 93% and 79% for 30-day, 60-day and 1-year readmissions<br>→ The study does, however, not elaborate on potential economic impacts for the hospital when deploying the tool	Deployment of a CNN learning features from time series of vital signs and other data of real-world EHRs of a large US-based hospital's general hospital ward and operating room data	USA	→ Advanced Analytics<br>→ Indirect positive economic impact
**[66] Almeida et al. (2016): A disruptive Big data approach to leverage the efficiency in management and clinical decision support in a Hospital**	→ Presents an analytics platform based on Big Data analysis via Machine Learning analyzing various sources of clinical information incl. EHRs to improve outcomes and efficiency	→ The System showed potential to increase efficiency by predicting 30% of intensive care unit (ICU) admissions and 50% of non-ICU inpatient deaths<br>→ Nevertheless, the authors only hypothesize this efficiency improvement potential	Qualitative case study of the São João Hospital Center	Portugal	→ Advanced Analytics<br>→ Indirect positive economic impact
**[67] Peck et al. (2014): Characterizing the value of predictive analytics in facilitating hospital patient flow**	→ Examines the impact of sharing predictions on the patient flow from emergency department (ED) to inpatient units (IU) with physicians by using discrete event simulation predictive analytics	→ Sharing information on crowding levels and total expected bed needs resulted in a statistically significant reduction in boarding time from the ED to IUs between 11.69% and 18.38% (dependent on hospital type) ($p<0.05$)	Discrete event simulation predictive analytics based on current emergency department information	USA	→ Advanced Analytics<br>→ Indirect positive economic impact
**[68] Stekel et al. (2019): Use of Image-Based Analytics for Ultrasound Practice Management and Efficiency Improvement**	→ Examines the advantages of a business/workflow analytics tool of an ultrasound practice in terms of equipment utilization and potential levers for efficiency improvements	→ Probe utilization data was used to support purchasing decision making resulting in not replacing broken probes and thereby saving $10,000<br>→ Scan time analysis resulted in a short-list of exams that would benefit the most from efficiency improvements<br>→ Physician efficiency analysis and benchmarking was seen valuable for best practice identification	Statistical analysis of an undisclosed number of ultrasound images and related metadata and procedure data such as exam length	USA	→ Business Analytics<br>→ Direct positive economic impact
**[70] Dulac et al. (2017): A data-driven approach to improving clinical care and reducing costs**	→ Presents two examples of how data analytics can reduce costs in health systems	→ UTMB was able to reduce penalty payment reductions to 0% by identifying imprecise EHR documentation and coding for two key	Qualitative case studies of UTMB Health, Texas and Franciscan Alliance, Indiana	USA	→ Business Analytics

Study	Objective	Findings	Methodology & data	Region	Categorization
	→ UTMB Health (UTMB) used data analytics to uncover root causes of high preventable complication and readmission rates which implied penalty payment reductions totaling 3.5% of all payments<br>→ Franciscan Alliance (FA) used data analytics to identify best-and worst-performing physicians to align performances, improve quality and save costs	obstetrical preventable complications and for a misinterpreted readmission reason<br>→ FA realized that best-performing physicians had $1,232 lower costs for each heart failure case treated compared to lowest performers; Based on these findings a new program to potentially achieve best-performer efficiency was introduced			→ Direct positive economic impact (UTMB)<br>→ Indirect positive economic impact (FA)
**[71] Rivera et al. (2015): Using business analytics to improve outcomes**	→ Presents the impact of several business analytics applications in the fields of physician performance and billing analytics at the Orlando Health Physician Group	→ Physician performance analytics: At the group, total contribution margins are calculated for every single provider to allow for benchmarking, target setting and tracking, however, an actual cost/efficiency-impact was not considered by the authors<br>→ Billing analytics: By prescreening patients for potential payment defaults, around $14 million per year are saved in bad debt write-offs according to the group	Qualitative case study of the Orlando Health Physician Group	USA	→ Business Analytics<br>→ Direct positive economic impact
**[72] Schouten, P. (2013): Big data in health care: solving provider revenue leakage with advanced analytics**	→ Presents the impact of a pattern-based business analytics solution to address revenue leakage problems of a health system	→ The health system reported to increase the probability of detecting missed charges and decrease audit-related costs<br>→ Overall, 2% of outpatient revenue that had previously gone unbilled was identified and audit costs were reduced by 75%<br>→ The author claims that based on industry average, hospitals deploying analytics solutions are able to increase operating income by 10%	Qualitative case study of a large US-based health system	USA	→ Business Analytics<br>→ Direct positive economic impact

Study	Objective	Findings	Methodology & data	Region	Categorization
**[73] Stoves et al. (2010): Electronic consultation as an alternative to hospital referral for patients with chronic kidney disease: a novel application for networked electronic health records to improve the accessibility and efficiency of healthcare**	→ Examines the advantages of an electronic medical round in the field of chronic kidney care connecting general practitioners (GP) with nephrologists in the UK	→ Electronic consultations between GPs and nephrologists resulted in an increase of 34%-points of referrals being considered as appropriate compared to paper-based communication (based on expert opinion) and in 78%-points fewer referrals to the hospital<br>→ Physician and nephrologist interviews and questionnaires revealed perceived efficiency improvements following the introduction	Quantitative comparison of patterns and quality between paper-based and electronic referrals based on 12-month referral data from 85 practices; Qualitative analysis of physician experiences in terms of efficiency and quality based on phone interviews and questionnaires	United Kingdom (UK)	→ Telemedicine<br>→ Indirect positive economic impact
		**Targeted supplementary literature search (n=7)**			
**[5] Bright et al. (2012): Effect of clinical decision-support systems: a systematic review**	→ Presents a systematic review and meta-analysis of the effects of clinical decision-support (CDS) systems on clinical outcomes, care processes, workload and efficiency, patient satisfaction, costs and provider use	→ The authors observed limited evidence for benefits of CDS for clinical, economic, workload and efficiency outcomes<br>→ Significant positive impact could be shown for process measures such as better choices in the preventive care services, appropriate clinical studies or treatments ordered/prescribed<br>→ Modest positive impact could be shown for effects on morbidity with potential chain-effects for mortality<br>→ Modest evidence for a positive impact on costs was shown based on 22 studies	Systematic review and meta-analysis based on a Medline search for studies between 1976 and January 2011 including 148 unique studies	Various	→ Clinical Decision Support<br>→ Direct positive economic impact
**[38] Zlabek et al. (2011): Early cost and safety benefits of an inpatient electronic health record**	→ Examines the effect of EHR and computerized physician order entry (CPOE) implementation on quality, cost and safety of care	→ Surrogates for quality were length of stay (LOS), 30-day readmissions, case mix and risk-adjusted mortality<br>→ Surrogates for costs were laboratory tests, radiology examinations, transcription costs and paper consumption<br>→ No significant effect of the EHR or CPOE was observed on the quality surrogates<br>→ The number of laboratory tests showed no significant change right after the EHR introduction, however, decreased by 18%	Pre-post statistical analysis based on data collected from 1 year before to 1 year after EHR introduction (CPOE system was introduced 3 months after EHR introduction) at the 325-bed Gundersen Lutheran Medical Center	USA	→ Electronic Health Records<br>→ Direct positive economic impact

Study	Objective	Findings	Methodology & data	Region	Categorization
		from pre-EHR to 9 months post-CPOE introduction (p<0.001)<br>→ A similar pattern was observed for the number of radiology examinations with a 6.3% decrease after CPOE introduction (p<0.009)<br>→ Monthly transcription costs decreased by 74.6% right after EHR introduction resulting in $667,896 savings in the year after EHR introduction (p<0.001)<br>→ Paper savings totaled $30,531 in the year after EHR introduction (p<0.001)			
**[52] McDowell et al. (2017): Effect of the implementation of a new electronic health record system on surgical case turnover time**	→ Examines the impact of an EHR introduction on the efficiency of the operating room in terms of surgical case turnover time (TOT) at a US-based hospital	→ EHR implementation led to a significant efficiency decrease in the operating room persisting over a 5-month time period<br>→ TOT increased on average by 10 min (63 vs 53, p<0.001) in the first month after implementation, which slightly improved in the second (59 vs. 53, p<0.001) and remained at the pre-implementation baseline starting 5 months after EHR implementation	Statistical pre-post analysis of mean TOTs covering 25,499 cases divided into a pre- and post-implementation group	USA	→ Electronic Health Records<br>→ Indirect negative economic impact
**[58] Levick et al. (2013): Reducing unnecessary testing in a CPOE system through implementation of a targeted CDS intervention**	→ Examines the impact of introducing a clinical decision support (CDS) intervention, i.e. an alert for B-Type Natriuretic Peptide (BNP) testing, in a computerized physician order entry (CPOE) system, on test volume and costs	→ The introduction of the CDS alert resulted in a significant reduction of tests of 21% relative to the mean, ultimately saving an estimated $92,000 per year	Multiple regression analysis based on a sample of 41.306 patients with at least one BNP test at the provider network between January 2008 and September 2011	USA	→ Clinical Decision Support<br>→ Direct positive economic impact
**[64] Trivedi et al. (2018): Automatic determination of the need for intravenous contrast in musculoskeletal MRI examinations using IBM Watson's natural**	→ Examines the potential of using the IBM Watson Natural Language Processing (NLP) algorithm to automatically assign usage of intravenous contrast for	→ Watson correctly assigned 129 of 140 "with contrast" (WC) protocols achieving a sensitivity of 0.743, a specificity of 0.921, a positive predictive value of 0.904 and an overall accuracy of 0.832<br>→ The authors hypothesize that the utilization of this decision-support tool may increase	Application of 8 traditional machine learning models and IBM Watson's deep learning-based NLP model to a dataset of 1,520 MRI exams (1,240 training set, 280 test set)	USA	→ Advanced Analytics<br>→ Indirect positive economic impact

Study	Objective	Findings	Methodology & data	Region	Categorization
**language processing algorithm**	musculoskeletal magnetic resonance imaging (MRI) during protocol creation based on free-text EHR information	efficiency for radiologist, however, do not provide any quantitative proof			
**[69] Swedberg, C. (2013): RFID boosts medical equipment usage at UK hospital**	→ Presents the effect of introducing a radio-frequency identification (RFID) system to track loaned medical equipment on equipment utilization and lending costs	→ Equipment utilization rates increased from 5% to 40% reducing the need to rent or purchase equipment saving an estimated $200,000 per year	Qualitative case study of a UK-based 1,100-bed hospital	UK	→ Business Analytics<br>→ Direct positive economic impact
**[74] Heidbuchel et al. (2015): EuroEco (European Health Economic Trial on Home Monitoring in ICD Patients): a provider perspective in five European countries on costs and net financial impact of follow-up with or without remote monitoring**	Examines differences in costs and financial impact between remote and in-office follow-ups (FU) for implantable cardiac defibrillators (ICD) for providers, as well as, for healthcare payers	→ Remote FUs resulted in fewer in-person visits (3.79 +/-1.67 vs. 5.53 +/-2.32; p<0.001), more non-office-based contacts (1.95 +/-3.29 vs. 1.01 +/-2.64; p<0.001), more internet sessions (11.02 +/-15.28 vs. 0.06 +/-0.31; p<0.001), as well as, fewer hospitalizations and shorter length-of-stay (last 2 not statistically significant)<br>→ On average, the total cost and net financial impact for providers did not show differences between remote and in-office FUs (Mean (95% CI): €204 (169–238) vs. €213 (182–243), not significant)<br>→ Importantly, however, regional heterogeneity could be observed where providers in countries with remote FU reimbursement in place showed maintained or increased profit, whereas in other countries less profit was achieved	Randomized, non-blinded, parallel-design trial in 17 provider centers from six European countries (Belgium, Finland, Germany, UK, Spain & Netherlands) covering 242 patients completing the study	Europe	→ Telemedicine<br>→ Neutral economic impact
